# Phenotypic Differentiation Within the *aac(6*′*)* Aminoglycoside Resistance Gene Family Suggests a Novel Subtype IV of Contemporary Clinical Relevance

**DOI:** 10.3390/antibiotics13121196

**Published:** 2024-12-08

**Authors:** Michel Plattner, Maurizio Catelani, Sarah-Lisa Gmür, Maximilian Hartmann, Fatmanur Kiliç, Klara Haldimann, David Crich, Sven N. Hobbie

**Affiliations:** 1 Institute of Medical Microbiology, University of Zurich, 8006 Zurich, Switzerland; 2 Department of Pharmaceutical and Biomedical Sciences, University of Georgia, Athens, GA 30602, USA; 3 Division of Clinical Bacteriology and Mycology, University Hospital Basel, 4031 Basel, Switzerland

**Keywords:** aminoglycoside antibiotics, acetyltransferases, whole genome sequencing, resistance gene annotation, surveillance, phenotype prediction, in silico antimicrobial susceptibility testing

## Abstract

Background: Whole genome sequencing of clinical bacterial isolates holds promise in predicting their susceptibility to antibiotic therapy, based on a detailed understanding of the phenotypic manifestation of genotypic variation. The *aac(6*′*)* aminoglycoside acetyltransferase gene family is the most abundant aminoglycoside resistance determinant encountered in clinical practice. A variety of AAC(6′) isozymes have been described, suggesting a phenotypic distinction between subtype I, conferring resistance to amikacin (AMK), and subtype II, conferring resistance to gentamicin (GEN) instead. However, the epidemiology and thus clinical relevance of the various and diverse isozymes and their phenotypic distinction demand systematic and contemporary re-assessment to reliably predict bacterial susceptibility to aminoglycoside antibiotics. Methods: We analyzed the resistance gene annotations of 657,603 clinical bacterial isolates to assess the prevalence and diversity of *aac(6*′*)* genes. Seventeen unique *aac(6*′*)* amino acid sequences were cloned and expressed under defined promoter control in otherwise isogenic *E. coli* cells for phenotypic analysis with twenty distinct aminoglycoside antibiotics. A panel of clinical isolates was analyzed for the genotype–phenotype correlation of *aac(6*′*)*. Results: An *aac(6*′*)* resistance gene annotation was found in 139,236 (21.2%) of the clinical isolates analyzed. AMK resistance-conferring *aac(6*′*)-I* genes dominated in *Enterobacterales* (28.5%). In *Pseudomonas aeruginosa* and *Acinetobacter baumannii*, a gene conferring the *aac(6*′*)-II* phenotype but annotated as *aac(6*′*)-Ib*_4_ was the most prevalent. None of the *aac(6*′*)* genes were annotated as subtype III, but gene *aac(6*′*)-Ii* identified in Gram-positive isolates displayed a subtype III phenotype. Genes that were annotated as *aac(6*′*)-Ib*_11_ in *Enterobacterales* conferred resistance to both AMK and GEN, which we propose constitutes a novel subtype IV when applying established nomenclature. A phenotypic assessment facilitated structural re-assessment of the substrate promiscuity of AAC(6′) enzymes. Conclusions: Our study provides the most comprehensive analysis of clinically relevant *aac(6*′*)* gene sequence variations to date, providing new insights into a differentiated substrate promiscuity across the genotypic spectrum of this gene family, thus translating into a critical contribution towards the development of amino acid sequence-based in silico antimicrobial susceptibility testing (AST).

## 1. Introduction

Aminoglycoside antibiotics play an important role in the treatment of serious systemic infections such as blood stream infections (BSI), endocarditis, pneumonia, pyelonephritis, and other complicated urinary tract infections [1]. Bacterial resistance to aminoglycosides has therefore been of concern. The most abundant aminoglycoside resistance genes belong to the *aac(6*′*)* gene family coding for an aminoglycoside acetyltransferase AAC(6′) that acetylates the 6′-amino group of 4,6-disubstituted 2-deoxystreptamines such as gentamicin (GEN), tobramycin (TOB), or amikacin (AMK) (reviewed in [2,3]).

The genotypic variety of *aac(6*′*)* genes and the functional differentiation of AAC(6′) enzymes has been described in numerous studies in the past three decades. A structural analysis revealed that AAC(6′) functionality originates through the convergent evolution from diverse Gcn5-related-*N*-acetyltransferase (GNAT) ancestral enzymes [4], with amino acid sequence homologies suggesting a phylogenetic tree branching into three distinct clades of AAC(6′) enzymes [5].

Functional classification and subtyping of aminoglycoside modifying enzymes was adopted long before gene sequencing became a routine tool in the microbiology laboratory and has nevertheless retained its clinical utility in the susceptibility profiling of drug-resistant clinical isolates. As a result of this, the diversity of *aac(6*′*)* genes has been functionally differentiated by its phenotypic resistance profile instead of its phylogenetic association [6,7]. While the functional differentiation can be reflective of the sequence homology of aminoglycoside resistance genes, as it has been described for the *aac*(*3*) family [8], this is not the case for the *aac(6*′*)* family.

Historically, the functional differentiation by substrate specificity has divided the AAC(6′) enzymes into two distinct groups. Subtype I confers resistance to TOB and AMK, but not to GEN. Subtype II confers resistance to TOB and GEN, but not to AMK [6,7]. More recently, a subtype III has been proposed to create a category for an *aac(6*′*)* resistance gene that confers resistance to TOB only, but neither to AMK nor to GEN [9].

While the discrepancy between sequence homology and phenotypic manifestation has already posed a challenge for the sequence-homology-based prediction of bacterial susceptibility to specific aminoglycosides, the increase in genome sequencing has only further raised our awareness of the complexity around genetic diversity of resistance genes.

In the present study, we aimed to consolidate the current knowledge around phenotypic manifestation of *aac(6*′*)* genotypic diversity, with a clear focus on clinical utility and potential guidance in the computational prediction of aminoglycoside susceptibility profiles. First, we analyzed the NCBI National Database of Antibiotic Resistant Organisms (NDARO) whole genome repository for the contemporary abundance, diversity, and distribution of individual *aac(6*′*)* resistance gene annotations. We then curated the phylogenetic relationship of the different AAC(6′) protein sequences and determined the associated phenotypic resistance profiles, leading us to propose a new AAC(6′) subtype IV that confers resistance to TOB, AMK, and GEN.

## 2. Results

### 2.1. Diversity and Epidemiology of aac(6′) Genes

**Figure 1:**
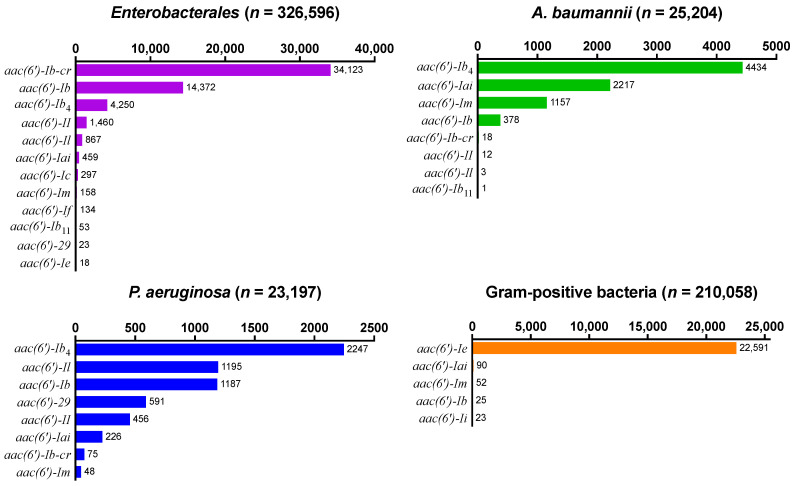
Clinical relevance of individual *aac(6*′*)* genes. Prevalence of *aac(6*′*)* gene annotations in 657,603 bacterial clinical isolates deposited in the NDARO on 1 July 2024. Gene and protein names were assigned according to the NDARO reference gene catalogue.

First, we analyzed the resistance gene annotations and their corresponding amino acid sequences for 657,603 clinical bacterial isolates to learn more about the abundance and distribution of *aac(6*′*)* genes. In *Enterobacterales*, *aac(6*′*)-Ib-cr* was the most prevalent resistance gene (*n* = 34,123; 10.4%), followed by *aac(6*′*)-Ib* (*n* = 14,372; 4.4%) and *aac(6*′*)-Ib*_4_ (*n* = 4250; 1.3%) (Figure 1). In contrast, *aac(6*′*)-Ib*_4_ was the most prevalent *aac(6*′*)* gene in *A. baumannii* (*n* = 4434: 17.6%) and *P. aeruginosa* (*n* = 2247; 9.7%), and *aac(6*′*)-Ib-cr* was rare in these non-fermenters. In Gram-positive isolates, *aac(6*′*)-Ie* (*n* = 22,591; 10.8%) was the only relevant *aac(6*′*)* gene. Across all the bacterial genomes and species studied, subtype II was relatively rare in comparison to subtype I, and none of the genes were annotated as subtype III (Figure 1).

**Figure 2:**
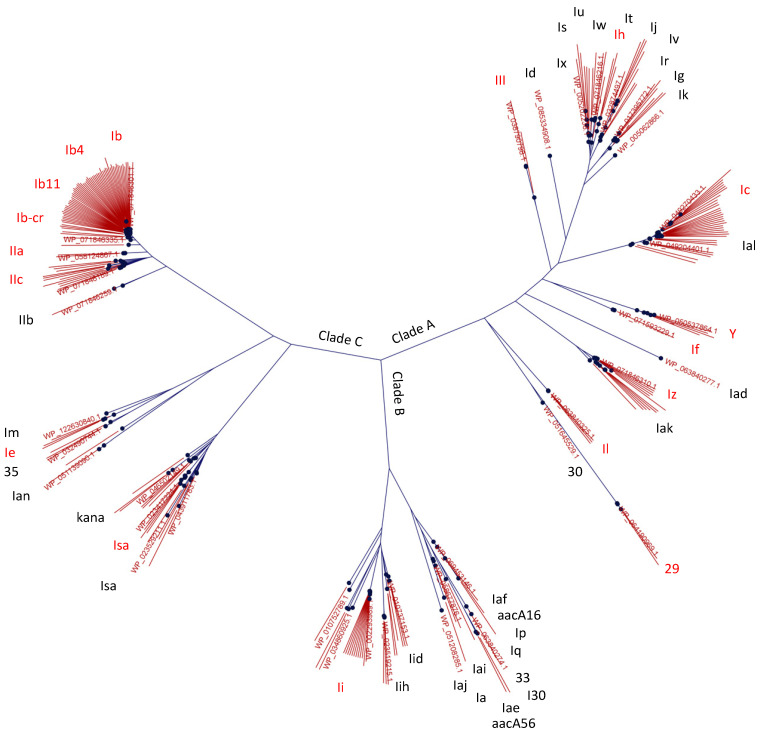
Amino acid sequence homology of individual *aac(6*′*)* genes. Unrooted phylogenetic tree of AAC(6′) amino acid sequences depicting the homology between clinically relevant isozymes and other subtypes previously described. Genes subcloned for phenotypic analysis are indicated in red font.

Visualizing the amino acid sequence homologies of the 264 distinct AAC(6′) enzymes present in the NDARO reference gene catalog at the time of analysis resulted in a phylogenetic tree with three major clades A, B, and C (Figure 2), similar to a previously reported tree that was based on only a few sequences [5]. Based on a pre-existing annotation of the reference genes alone, the tree suggests subtype AAC(6′)-I to be present in all three clades, whereas subtype II locates exclusively in clade C, and subtype III and *Yersinia*-associated AAC(6′) exclusively in clade A. The distribution of subtype I across the three clades may not be surprising considering a nomenclature that, for clinical practicability, has traditionally classified aminoglycoside-modifying enzymes based on phenotypic manifestation rather than protein homology.

### 2.2. Phenotypic Differentiation of aac(6′) Gene Variants Recombinantly Expressed in Engineered E. coli Strains

**Figure 3:**
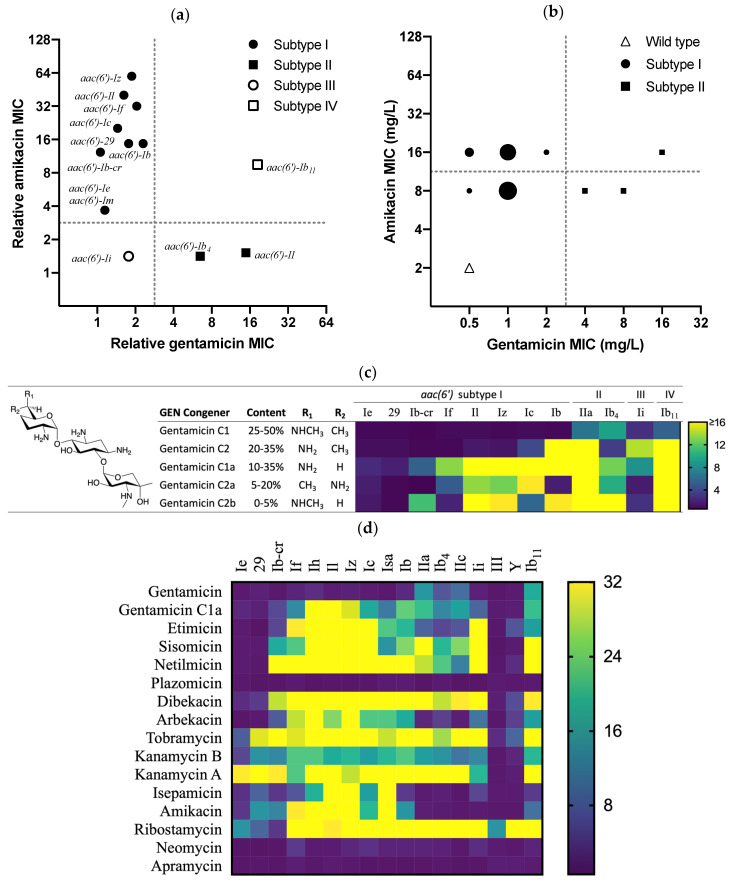
Phenotypic assessment of *aac(6*′*)* genes in engineered strains and clinical isolates. (**a**) Subtyping of representative *aac(6*′*)* genes by the changes in susceptibility to AMK and GEN relative to the wild-type (WT) laboratory strain DH5α. An interpretative threshold of > 2-fold increase in MIC is indicated by dotted lines; (**b**) manifestation of the corresponding phenotypes in clinical isolates not expressing aminoglycoside-modifying enzymes other than *aac(6*′*)*. The size of the symbols corresponds to the number of isolates, and clinical breakpoints are indicated by dotted lines. (**c**,**d**) Heat map visualization of the differential activity of *aac(6*′*)* gene variants for individual gentamicin congeners (**c**) and fifteen additional aminoglycoside antibiotics (**d**) displayed as fold-increase over wild-type MIC.

To elucidate the substrate promiscuity of individual AAC(6′) variants, we cloned distinct *aac(6*′*)* gene sequences for heterologous expression under defined promoter control in the laboratory strain *E. coli* DH5α. Constitutive promoter control was assessed and confirmed phenotypically (Table S1). Nine of the cloned *aac(6*′*)* genes including *aac(6*′*)-I* and *aac(6*′*)-Ib* resulted in a decreased susceptibility to AMK but not to GEN, which is indicative of a phenotypic classification as subtype I (top left quadrant in Figure 3a). Two of the genes resulted in a decreased susceptibility to GEN but not AMK, allocating the two genes to subtype II, including a gene generally misannotated as subtype I variant *aac(6*′*)-Ib*_4_ (bottom right quadrant in Figure 3a). The *aac(6*′*)-Ii* gene decreased susceptibility to TOB but did not affect the susceptibility to either AMK or GEN, assigning it phenotypically to subtype III (bottom left quadrant in Figure 3a). We found the *aac(6*′*)-Ib*_11_ gene to decrease the susceptibility to both AMK and GEN, in addition to TOB (top right quadrant in Figure 3a). This corresponds to a substrate promiscuity not observed for any of the other *aac(6*′*)* genes, and a phenotype incompatible with the definitions laid out for subtypes I, II, and III. The phenotypic differentiation of subtypes is summarized in Table 1.

### 2.3. Phenotypic Assessment in Clinical Enterobacterales Isolates

Using engineered strains is an advantage in studying the effect of individual genes in a defined system and against an isogenic background. However, it is not necessarily indicative of the phenotypic manifestation of these genes in clinical isolates. We therefore analyzed genome sequences to identify 20 *E. coli* bacterial clinical isolates with a confirmed presence of an *aac(6*′*)* gene, and a concurrent absence of any other aminoglycoside resistance gene that could obscure our phenotypic analysis. Of these, 17 were found to be of subtype I phenotype, and the other three of a subtype II phenotype (Figure 3b). Closer inspection of the 20 AAC(6′) amino acid sequences revealed that the three isolates with a subtype II phenotype each carried an *aac(6*′*)-Ib*_4_ gene, whereas the other 17 contained either an *aac(6*′*)-Ib-cr* or an *aac(6*′*)-Ia* gene variant (Table S2). 

### 2.4. Mechanistic Analysis Using Other Aminoglycoside Antibiotics

GEN plays a pivotal role in the phenotypic subtype manifestation for any given *aac(6*′*)*. However, GEN does not comprise a single chemical entity but rather a mixture of gentamicin C congeners, prompting us to study the substrate specificity of the various AAC(6′) enzymes for individual purified gentamicin C congeners. Gentamicin C1, C6′,N6′-dimethylated, and the primary constituent in clinical GEN mixtures were the congeners most recalcitrant to acetylation by AAC(6′). Only subtypes II and IV resulted in elevated gentamicin C1 MICs (Figure 3c), providing a possible rationale for the historic connotation of subtype I not causing resistance to GEN because it does not efficiently acetylate the predominant constituent in the GEN mixture. In contrast, the MICs of the four minor GEN congeners were additionally affected by the subtype I and III variants, albeit to different extents. The MIC of gentamicin C1a, the only congener without methylation around the 6′ position, was found to be most affected by AAC(6′), indicating it was most amenable to 6′-*N*-acetylation and therefore inactivation. The *aac(6*′*)-Ib*_11_ gene resulted in some of the highest MICs of all GEN congeners (Figure 3c). The differential activity of subtype II versus subtype I across individual GEN congeners was also confirmed with two clinical isolates each (Table S3).

Of the thirteen 4,6-disubstituted 2-dexoystreptamine aminoglycosides tested, only GEN and plazomicin are protected from acylation by AAC(6′) activity via substitution at one or both of the C6′ and N6′ positions (chemical structures are provided for reference in Figure 3c and Figure S1). The free 6′-amino groups of AMK, TOB, netilmicin, etimicin, dibekacin, arbekacin, sisomicin, isepamicin, kanamycin A, and kanamycin B are subject to acetylation by at least some of the AAC(6′) subtypes (Figure 3d). The 1-*N*-acylations in AMK, arbekacin, and isepamicin are known to preserve the antibacterial activity of these molecules in the presence of aminoglycoside-modifying enzymes, and the AAC(6′) of subtype II were no exception. However, the activity of these three molecules was reduced by subtype I isozymes, albeit to a lesser extent than their parent molecules kanamycin A and dibekacin that lack the 1-*N*-substitutions (Figure 3d). Protection from AAC(6′) activity by the 1-*N*-ethylations in etimicin and netilmicin in comparison to their parent compounds gentamicin C1a and sisomicin was found to be minor at best.

The 4,5-disubstituted 2-deoxystreptamine ribostamycin appeared to be the most broadly susceptible substrate as it showed little activity in the presence of the spectrum of AAC(6′) subtypes, including the least promiscuous subtypes III, Ie, and Y (the *Yersinia* variant, Figure 3d). This phenotypic observation contrasted significantly with that for neomycin, which differs from ribostamycin by the presence of a third aminosugar residue with an additional two basic amino groups (Figure S1). The susceptibility of *E. coli aac(6*′*)* strains to neomycin was reduced by not more than 2- to 8-fold relative to the wild-type strain (Figure 3d).

Apramycin lacks a 6′-amino group and is therefore intrinsically protected from AAC(6′) resistance (Figure 3d).

## 3. Discussion

Our study findings underscore the need to assess, define, and curate the phenotypic manifestation of individual resistance gene variants encountered in clinical practice carefully and continuously. While for the *aac(6*′*)* gene family alone, there has already been knowledge of three distinct aminoglycoside resistant patterns, we propose to define a novel subtype IV that may be seen as particularly concerning on the basis of conferring the least discriminate aminoglycoside resistance, compromising the activity of all three aminoglycoside antibiotics widely used in the clinics: AMK, GEN, and TOB.

Decreased susceptibility to AMK appears to be the predominant *aac(6*′*)*-associated phenotype in *Enterobacterales*, primarily encoded by the two gene variants *aac(6*′*)-Ib-cr* and *aac(6*′*)-Ib* (Figure 1). A W87R substitution in *aac(6*′*)-Ib-cr* results in additional fluoroquinolone resistance and may have therefore been enriched over time by additional selection pressures [10]. These findings are in line with the common perception around *aac(6*′*)* [11] but provide unprecedented quantitative evidence. In *A. baumannii* and *P. aeruginosa*, however, decreased susceptibility to GEN by means of an *aac(6*′*)-Ib*_4_-encoded subtype II phenotype [12,13] appears to be the more abundant manifestation (Figure 1). Tobramycin, an aminoglycoside preferentially used in the treatment of *P. aeruginosa* infections, is inactivated by both subtypes I and II. *aac(6*′*)-29* was another relatively abundant resistance gene in *P. aeruginosa* in which it was first reported [14] and based on its resistance profile to be classified as subtype I (Figure 3d). Gene variant *aac(6*′*)-Ib*_11_ confers resistance to AMK, GEN, and TOB [12,15], which we propose constitutes a novel subtype IV distinct from subtypes I, II, and III based on the resistance pattern it confers (Figure 3a). Albeit still rare at the time of analysis and primarily confined to *Enterobacterales*, its concerning phenotype may warrant continued surveillance of the evolving epidemiology of this specific gene variant. An *aac(6*′*)-III* gene previously discovered in *Burkholderia* and labelled as distinct subtype III for its more stringent substrate specificity when compared to subtypes I and II [9] was not identified in nearly 660,000 of the clinically most relevant bacterial pathogens studied here (Figure 1) and shares little protein homology with any of the more frequently annotated genes (Figure 2). However, we found that the *aac(6*′*)* genes of clade B such as *aac(6*′*)-Ii* may phenotypically be interpreted as subtype III as well, since synthesis and cloning of this gene resulted in a decreased susceptibility to TOB, but neither AMK nor GEN (Figure 3d). This limited protection and the fact that it is a chromosomal gene in *Enterococcus faecalis* lead to speculations that it has not primarily evolved as an antibiotic resistance gene [16]. Indeed, Clade B gene annotations were scarce in the studied bacterial populations and limited to a small number of Gram-positive bacteria (Figure 1). In a separate analysis for *Neisseria gonorrhoeae*, no *aac(6*′*)* resistance gene annotations were found, which is in agreement with the complete absence of relevant levels of aminoglycoside resistance in gonococci highlighted previously [17].

**Figure 4:**
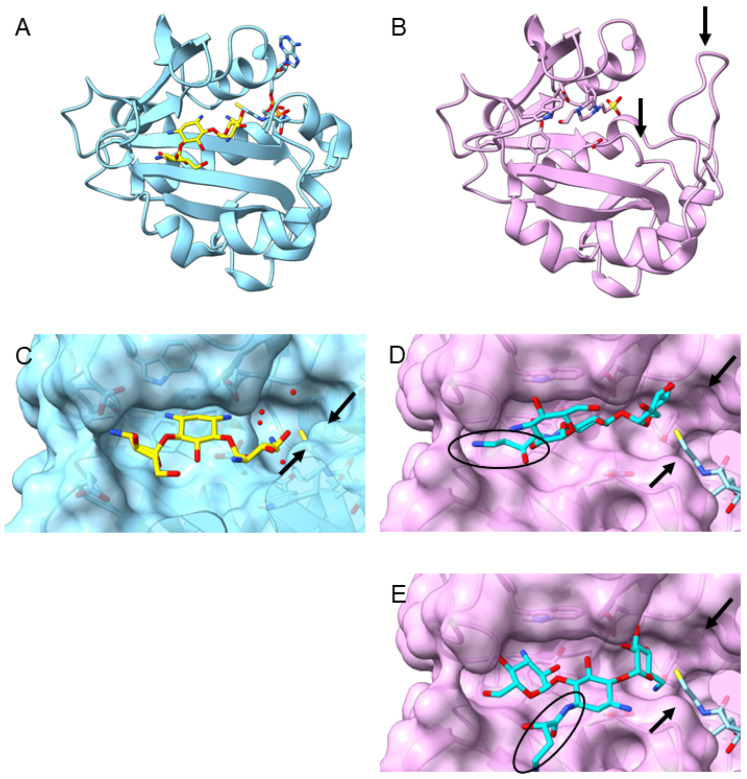
Structural comparison of AAC(6′)-Ib_11_ with AAC(6′)-Ib. (**A**) Ribbon structure of AAC(6′)-Ib with bound kanamycin B (yellow sticks) and co-enzyme A (PDB ID: 2qir); (**B**) ribbon structure of AAC(6′)-Ib_11_ (PDB ID: 2pr8), showing the loop rearrangement (arrows) by amino acid substitutions Q101L, L102S relative to AAC(6′)-Ib, which results in an extended substrate promiscuity; (**C**) closeup view of kanamycin B (yellow sticks) and co-enzyme A (covered) in the substrate-binding pocket of AAC(6′)-Ib (represented as surface structure), highlighting the steric limitations for 1-*N*-substitutions superimposed onto kanamycin; (**D**,**E**) closeup views of two possible amikacin orientations (cyan sticks) modelled into the substrate-binding pocket of AAC(6′)-Ib_11_ using AutoDock Vina. The 2-deoxystreptamine ring of amikacin needs to be rotated around the O4-C1′-glycosidic bond when compared to kanamycin to permit accommodation of its 1-*N*-LHABA substitution marked by the black circles. The loop rearrangement in AAC(6′)-Ib_11_ results in a cleft (indicated by black arrows) in the binding pocket of co-enzyme A (sky blue), suggesting a wider space than in AAC(6′)-Ib available to better accommodate the 6′-disubstitutions of gentamicin C1 and C2a.

In the *aac(6*′*)-Ib* gene family, individual amino acid substitutions are sufficient to result in changes in phenotypic manifestation. Our data for contemporary clinical variants are therefore in good agreement with a previous analysis by the Tolmasky lab [18]. The increased substrate promiscuity observed for the gene variant *aac(6*′*)-Ib*_11_ has been reported previously and rationalized by a loop formation that may be indirectly triggered by the Q101L substitution [12]. However, the structural rationale provided by the authors was based on co-crystallization of kanamycin B and apparently inadequate. Attempts to co-crystallize the AAC(6′)-Ib protein with AMK instead failed [15]. The proposition that AMK can be superimposed onto the kanamycin structure because the protein structure has sufficient space to accommodate a 3-*N*-substitution is flawed by the fact that the difference between kanamycin B and AMK is a 1-*N*-substitution instead, and the published structure of the kanamycin co-crystal would not permit accommodation of any bulkier 1-*N*-modification (Figure 4). Furthermore, AMK is a kanamycin A derivative and thus differs from kanamycin B additionally by its 2′-amino group (Figure S1). Interestingly, kanamycin A appears to be more effectively inactivated by AAC(6′) than kanamycin B (Figure 3d). Presumably, the loss of one of four basic amino groups in kanamyic A on 6′-*N*-acetylation has a more profound effect on its net positive charge and affinity for the ribosome binding than the parallel loss of one of five basic amines in kanamycin B [19]. Indeed, kanamycin A is generally more susceptible to structural modifications than the more highly positively charged kanamycin B [20,21]. It is also possible that the additional basic amino group at the 2′-position of kanamycin B limits its affinity for the AAC(6′) substrate binding pocket, hence reducing its rate of acetylation. Alternatively, since there is evidence for the 6′-acetylation of AMK by the AAC(6′)-Ib family of enzymes, we suggest that the 2-deoxystreptamine ring of AMK may be positioned differently than the co-crystallized kanamycin B suggests. This postulate is supported by docking studies that reveal two possible orientations of AMK that keep the 6′-amino group near the CoA (Figure 4D,E).

For the discriminate binding of gentamicin C1 and C2a to AAC(6′)-Ib_11_ but not to AAC(6′)-Ib, it is conceivable to assume the space around CoA that is widened by the loop formation accommodates the bulkier 6′-disubstitutions in GEN (depicted in Figure 3c and Figure S1) more easily than the tighter binding pocket in AAC(6′)-Ib (Figure 4C–E). Our results suggest that the inability of subtype AAC(6′)-I enzymes to inactivate GEN is due to their inability to inactivate the two major constituents gentamicin C1 and C2, whereas the other three congeners without a methyl group in position R_2_ are inactivated by subtype I, II, and IV enzymes (Figure 3c). This further corroborates our prior assumption that the C6′-methyl group bears the primary responsibility for protecting gentamicin C1 from inactivation [22] and that it is not the N6′-methyl that interferes with accommodation in the substrate binding pocket of AAC(6′)-I, as it has previously been suggested [23]. We further noticed the susceptibility pattern of the gentamicin C congeners reported for the gene labelled *aac(6*′*)-Ib* [23] is different from that of our *aac(6*′*)-Ib* strain but resembles that of our *aac(6*′*)-Ic* strain instead. This is likely again due to the prevailing inaccuracy in discriminating *aac(6*′*)-I* isotypes in the available reference gene catalogs, as we discussed above. A phenotypic comparison between gentamicin C1a and dibekacin seems to further suggest that a kanosamine ring (as in dibekacin, but also tobramycin and amikacin, Figure S1) is more easily accommodated in the substrate-binding pocket of various AAC(6′) subtypes than the dimethylated garosamine rings in GEN (Figure 3d and Figure S1), providing another possible rationale for the differences between amikacin and isepamicin, which have already been observed previously [24].

The 4,5-disubstituted ribostamycin (Figure S1) stood out by being effectively inactivated by virtually all subtypes including AAC(6′)-III (Figure 3d) in stark contrast to the structurally related neomycin, which was the only aminoglycoside antibiotic with a free 6′-amine group that retained activity in the presence of all seventeen AAC(6′) variants tested (Figure 3d). The crystal structures of AAC(6′)-Ib in complex with ribostamycin (PDB ID: 2bue) and paromomycin (PDB ID: 2vqy) do not suggest any space constraints to accommodation of the additional ring in paromomycin and neomycin. Furthermore, we have previously demonstrated that synthetic 6′-*N*-acetyl-neomycin retains significant levels of antiribosomal and antibacterial activity comparable to that here for neomycin in the *aac(6*′*)* strains [25]. It is likely therefore that the N6′ acetylation of neomycin, because of the residual five basic amino groups, impacts its activity less than that of ribostamycin, which has only four basic amino groups to begin with, just as kanamycin B, with its five amino groups, is more tolerant of modification than the tetramine kanamycin A. Consistent with this rationale, ribostamycin antibacterial activity is generally found to be more vulnerable to medicinal chemistry modifications than that of neomycin [26,27].

Structural elucidation appears to be somewhat hampered by the fact that, although certain subtypes seem to preferentially accommodate either AMK or GEN, both drugs are inactivated far less efficiently than, for instance, TOB or kanamycin A, resulting in much higher resistance to these latter drugs than to the less cognate AMK or GEN (Figure 3d). This observation may also explain the apparent difficulties co-crystallizing AAC(6′) with GEN or AMK. In summary and from a drug discovery perspective, however, we conclude more broadly that C6′-methylation protects from inactivation by AAC(6′) subtypes I and III. A 1-*N*-substitution protects from subtypes II, III, and IV but not subtype I.

The fact that clinically relevant aminoglycosides are less efficiently inactivated than more cognate substrates raises the question of what level of substrate promiscuity is required to compromise therapeutic efficacy. We therefore tested a panel of select clinical isolates with an *aac(6*′*)* gene to assess whether the impact on aminoglycoside susceptibility resembles the observations derived from tightly controlled engineered strains. The panel size of clinical isolates was compromised by the fact that the majority of resistant clinical isolates carry other aminoglycoside resistance genes in addition to *aac(6*′*)*, potentially obscuring its phenotypic assessment. For the twenty isolates void of any other aminoglycoside resistance genes, the MIC of AMK and GEN was indeed elevated by 4- to 32-fold when compared to the wild-type reference strain. For about half the *aac(6*′*)-I* isolates studied, a fourfold decrease in susceptibility to AMK resulted in MICs that were covered by the clinical breakpoint of ≤8 µg/mL for AMK (Figure 3b), whereas an eightfold increase to 16 µg/mL would be considered AMK-resistant. This provides additional rationale for previous reports on aminoglycoside-“susceptible” *aac(6*′*)-Ib* isolates based on EUCAST or CLSI interpretative criteria [11,28]. In the small sample size studied, three isolates carried an *aac(6*′*)-Ib*_4_ gene, resulting in GEN resistance with MICs above the clinical breakpoint of ≤2 µg/mL (Figure 3b).

The limitation of this study corresponds to the limitations of the underlying database that was used to retrieve the data for analysis. The number of 657,603 genomes could be further increased in future studies by including additional databases in the analysis. However, this will require careful curation to avoid duplicate entries of genome sequences that have been deposited in more than one database. Furthermore, future studies could widen the scope to not just analyze genome annotations but also locate the resistance genes of interest to a specific genetic element in its host pathogen, providing additional insights with regards to differences in dissemination potential. Another limitation was the small number of clinical isolates identified for analysis that carry an *aac(6*′*)* gene but at the same time no other aminoglycoside resistance gene that would obscure phenotypic readout of aminoglycoside susceptibility in response to *aac(6*′*)*. A more comprehensive analysis may require access to much larger strain collections than were available to the authors at the time of this study.

## 4. Materials and Methods

### 4.1. Database Analysis for aac(6′) Resistance Genes

An epidemiologic database analysis for the genotypic susceptibility of clinical isolates was performed as described previously [8,29]. The data were downloaded from the NCBI National Database of Antibiotic Resistant Organisms (NDARO), https://www.ncbi.nlm.nih.gov/pathogens/antimicrobial-resistance (accessed on 1 July 2024). The inclusion criteria were “Isolation type: clinical”, “AMR genotypes: COMPLETE”, “Host: Homo sapiens, Homo sapiens sapiens, Homo_sapiens, “Homo sapiens”, “homo, homo sapiens sapiens, homosapiens, homo sapiens, Homosapiens”, and “property: has AMR genotype”. Resistance gene annotations for 657,603 genomes were analyzed for the presence of any of the 264 *aac(6*′*)* reference resistance genes (Table S4). The search included variants, subvariants, and mutants of all *aac(6*′*)* resistance gene families

A large number of sequences were only annotated with the generic term “*aac(6*′*)*” or “*aac(6*′*)-Ib*”. Therefore, a sequence alignment with Clustal Omega was performed, and a phylogenetic tree was generated in CLC Workbench 14. Clustering sequences were grouped, and unique annotations were used for the database analysis. The groups were as follows: *aac(6*′*)-Ib* (Ib, Ib′, Ib cr, Ib_3_, Ib_4_, Ib_11_, Ib cr3—Ib cr11), *aac(6*′*)-II* (II, IIa, IIb, IIc, 31, 32, aacA35, aacA47), *aac(6*′*)-Im* (Im, Ian, 35), *aac(6*′*)-Ic/aph*(*2*″) (Ia, If_2_), *aac(6*′*)-Isa* (Isa, kana), *aac(6*′*)-Ii* (Ii, Iid, Iih), *aac(6*′*)-Iai* (33, I30, Iae, Iaf, Iai, Iaj, aacA16, aacA56, Ia, Ip, Iq), *aac(6*′*)-29* (29, 29a, 29b, 30), *aac(6*′*)-Il*, *aac(6*′*)-Iz* (Iz, Iak), *aac(6*′*)-Iad*, *aac(6*′*)-If*, *aac(6*′*)-Ic* (Ic, Ial), *aac(6*′*)-Ig* (Ig, Ih, Ij, Ik, Ir, Is, It, Iu, Iv, Iw, Ix), and *aac(6*′*)-Id* (Id, III). The amino acid sequences of genes that were annotated as *aac(6*′*)-Ib* were aligned to further divide this gene family into 5 motifs differing from each other at amino acid positions 87, 101, 102, and 164 (Figures S2 and S3). A sequence alignment was used to assign the gene variants to three subcategories besides the genuine *aac(6*′*)-Ib* itself: *aac(6*′*)-Ib-cr* (Ib-cr, Ib-cr3, Ib-cr4, Ib-cr5, Ib-cr6, Ib-cr7, Ib-cr8, Ib-cr9, Ib-cr10 Ib-cr11), *aac(6*′*)-Ib*_4_ (Ib_4_, Ib_4_*, Ib′), and *aac(6*′*)-Ib*_11_ (Table S5).

### 4.2. Cloning of aac(6′) Resistance Genes

The engineered strains used in this study are summarized in Table S6. Individual *aac(6*′*)* gene sequences were synthesized (GeneArt Gene Synthesis, ThermoFisher, Reinach, Switzerland) and cloned into a low-copy-number plasmid. The genes were expressed under the control of an insulated constitutive promoter of defined strength as described previously [30]. The standard promoter strength of 0.03 was used for all experiments unless stated otherwise.

### 4.3. Identification and Selection of Clinical Isolates with an aac(6′) Resistance Gene Annotation

Clinical isolates carrying an *aac(6*′*)* gene were retrieved from a whole-genome sequenced collection of aminoglycoside-resistant *E. coli* clinical isolates described previously [31].

### 4.4. In Vitro Antimicrobial Susceptibility Testing

Minimal inhibitory concentrations (MICs) of antibiotics were determined by broth microdilution assays according to the European Committee on Antimicrobial Susceptibility Testing (EUCAST) reference method ISO 20776-1:2019 [32]. In brief, antibiotics were serially twofold diluted in cation-adjusted Mueller–Hinton broth, inoculated to a final cell density of 10^6^ CFU/mL, and incubated for 16–20 h at 35 ± 2 °C, followed by visual inspection for inhibition of bacterial growth. *E. coli* DH5α (ThermoFisher, Reinach, Switzerland) was used as a wild-type reference for the engineered strains, and *E. coli* ATCC 25922 was used as a quality control strain. The geometric mean of MICs was determined based on data from at least three independent experiments. Gentamicin C1, C1a, C2, and C2a were procured from TOKU-E (Sint-Denijs-Westrem, Belgium). Gentamicin C2b was synthesized by the Crich lab [22].

### 4.5. Substrate Docking Study

The SwissDock online interface [33] was used for a docking experiment with the amikacin ligand binding to AAC(6′)-Ib_11_. The applied docking method was AutoDock Vina with the following input parameters: “Ligand”, amikacin, provided as a SMILES string; “Target”, PDB ID 2pr8 chain B, all ligands removed; “Search parameters”, box center −5 Å, −33 Å, −16 Å; box size: 20 Å, 10 Å, 20 Å; and “Sampling exhaustivity”, 4. The docking results were analyzed using USCF ChimeraX [34]. Two possible ligand conformations were selected based on the relative positioning of the 6′-*N*-amino group of amikacin relative to the 6′-*N*-amino group of kanamycin B and to co-enzyme A in PDB ID 2qir.

## 5. Conclusions

Our study on the *aac(6*′*)* resistance gene family provides a case study for the critical importance of carefully curated WGS databases in combination with a detailed mechanistic understanding of susceptibility phenotypes associated with its underlying gene sequences. To develop WGS into a powerful and reliable method for the in silico prediction of drug susceptibility, it is important to carefully re-analyze and define the genotype–phenotype correlations for each resistance gene family at a resolution of individual amino acid residues. This will inevitably also shed additional light onto the molecular mechanisms of antibiotic resistance, providing valuable structure–activity relationships as starting points in rational drug design.

## Figures and Tables

**Table 1:** Simplified summary of the phenotypic differentiation between the four AAC(6′) subtypes.

AAC(6′) Subtype	GEN	AMK	TOB	Reference
I	S	R	R	[7]
II	R	S	R	[7]
III	S	S	R	[9]
IV	R	R	R	This study

S, susceptible; R, resistant.

## Data Availability

The original contributions presented in the study are included in the article/Supplementary Materials; further inquiries can be directed to the corresponding author.

## Supplementary Materials

The following supporting information can be collectively downloaded at https://www.mdpi.com/article/10.3390/antibiotics13121196/s1, Figure S1: Chemical structures of 2-deoxystreptamine aminoglycosides used in this study; Figure S2: Amino acid sequence alignment of AAC(6′)-Ib variants; Figure S3: Location of relevant amino acid substitutions in AAC(6′)-Ib variants; Table S1: Aminoglycoside susceptibility in response to AAC(6′) expressions levels in *E. coli* DH5α; Table S2: List of clinical isolates used in this study; Table S3: Gentamicin C MICs for clinical isolates of *aac(6*′*)* subtypes I and II; Table S4: Reference *aac(6*′*)* gene catalog; Table S5: Curation of the NDARO Reference Gene Catalog for *aac(6*′*)**-Ib*-like amino acid sequence homologies; Table S6: Engineered *E. coli* strains constructed in this study.
