# Supplementary material for: Phenotypic Differentiation Within the aac(6′) Aminoglycoside Resistance Gene Family Suggests a Novel Subtype IV of Contemporary Clinical Relevance
Source: Antibiotics (Basel). 2024 Dec 8;13(12):1196. doi: 10.3390/antibiotics13121196 (PMC11672645; doi:10.3390/antibiotics13121196)
Supplement: Supplementary file 1 [file antibiotics-13-01196-s001.zip › antibiotics-3348671-supplementary.pdf]

Phenotypic Differentiation Within the *aac(6′)* Aminoglycoside Resistance Gene Family Suggests a Novel Subtype IV of Contemporary Clinical Relevance

Michel Plattner [1], Maurizio Catelani [1], Sarah-Lisa Gmür [1], Maximilian Hartmann [1], Fatmanur Kiliç [1], Klara Haldimann [1], David Crich [2] and Sven N. Hobbie [1,3,*]

SUPPLEMENTARY MATERIALS

Table S1. Aminoglycoside susceptibility in response to AAC(6´) expressions levels in *E. coli* DH5α

	MIC (µg/mL)						
	**WT**	**AAC(6´)-I[b]**			**AAC(6´)-II[c]**		
Promoter[a]	**-**	**0.009**	**0.03**	**0.12**	**0.009**	**0.03**	**0.12**
Gentamicin	0.25-0.5	0.25-0.5	0.25-0.5	0.25	1-2	4-8	32
Amikacin	0.25-0.5	0.5-1	4	16	0.25	0.5	1
Tobramycin	0.5	4	16	16	8	16-32	64

[a] Relative promoter strength as defined in DOI: 10.1093/nar/gkq810

[b] AAC(6´)-I sequence WP_001749987

[c] AAC(6´)-II sequence WP_023622803

Table S2. List of clinical isolates used in this study with a confirmed *aac(6´)* gene and without any other aminoglycoside resistance gene that could obscure phenotypic analysis.

ID	Type	Amino acid sequence
1	Ib-cr	MSNAKTKLGITKYSIVTNSNDSVTLRLMTEHDLAMLYEWLNRSHIVEWWGGEEARPTLADVQEQYLPSVLAQESVTPYIAMLNGEPIGYAQSYVALGSGDG**R**WEEETDP<br>GVRGID**QL**LANASQLGKGLGTKLVRALVELLFNDPEVTKIQTDPSPSNLRAIRCYEKAGFERQGTVTTP**Y**GPAVYMVQTRFERTRSDA
2	Ib-cr	MSNAKTKLGITKYSIVTNSNDSVTLRLMTEHDLAMLYEWLNRSHIVEWWGGEEARPTLADVQEQYLPSVLAQESVTPYIAMLNGEPIGYAQSYVALGSGDG**R**WEEETDP<br>GVRGID**QL**LANASQLGKGLGTKLVRALVELLFNDPEVTKIQTDPSPSNLRAIRCYEKAGFERQGTVTTP**Y**GPAVYMVQTRQAFERTRSDA*
3	Ib-cr	MSNAKTKLGITKYSIVTNSNDSVTLRLMTEHDLAMLYEWLNRSHIVEWWGGEEARPTLADVQEQYLPSVLAQESVTPYIAMLNGEPIGYAQSYVALGSGDG**R**WEEETDP<br>GVRGID**QL**LANASQLGKGLGTKLVRALVELLFNDPEVTKIQTDPSPSNLRAIRCYEKAGFERQGTVTTP**Y**GPAVYMVQTRQAFERTRSDA*
4	Ib-cr	MSNAKTKLGITKYSIVTNSNDSVTLRLMTEHDLAMLYEWLNRSHIVEWWGGEEARPTLADVQEQYLPSVLAQESVTPYIAMLNGEPIGYAQSYVALGSGDG**R**WEEETDP<br>GVRGID**QL**LANASQLGKGLGTKLVRALVELLFNDPEVTKIQTDPSPSNLRAIRCYEKAGFERQGTVTTP**Y**GPAVYMVQTRQAFERTRSDA*
5	Ib-cr	MSNAKTKLGITKYSIVTNSNDSVTLRLMTEHDLAMLYEWLNRSHIVEWWGGEEARPTLADVQEQYLPSVLAQESVTPYIAMLNGEPIGYAQSYVALGSGDG**R**WEEETDP<br>GVRGID**QL**LANASQLGKGLGTKLVRALVELLFNDPEVTKIQTDPSPSNLRAIRCYEKAGFERQGTVTTP**Y**GPAVYMVQTRQAFERTRSDA*
6	Ib-cr	MSNAKTKLGITKYSIVTNSNDSVTLRLMTEHDLAMLYEWLNRSHIVEWWGGEEARPTLADVQEQYLPSVLAQESVTPYIAMLNGEPIGYAQSYVALGSGDG**R**WEEETDP<br>GVRGID**QL**LANASQLGKGLGTKLVRALVELLFNDPEVTKIQTDPSPSNLRAIRCYEKAGFERQGTVTTP**Y**GPAVYMVQTRQAFERTRSDA*
7	Ib-cr	MSNAKTKLGITKYSIVTNSNDSVTLRLMTEHDLAMLYEWLNRSHIVEWWGGEEARPTLADVQEQYLPSVLAQESVTPYIAMLNGEPIGYAQSYVALGSGDG**R**WEEETDP<br>GVRGID**QL**LANASQLGKGLGTKLVRALVELLFNDPEVTKIQTDPSPSNLRAIRCYEKAGFERQGTVTTP**Y**GPAVYMVQTRQAFERTRSDA*
8	Ib-cr	MSNAKTKLGITKYSIVTNSNDSVTLRLMTEHDLAMLYEWLNRSHIVEWWGGEEARPTLADVQEQYLPSVLAQESVTPYIAMLNGEPIGYAQSYVALGSGDG**R**WEEETDP<br>GVRGID**QL**LANASQLGKGLGTKLVRALVELLFNDPEVTKIQTDPSPSNLRAIRCYEKAGFERQGTVTTP**Y**GPAVYMVQTRQAFERTRSDA*
9	Ib-cr	MSNAKTKLGITKYSIVTNSNDSVTLRLMTEHDLAMLYEWLNRSHIVEWWGGEEARPTLADVQEQYLPSVLAQESVTPYIAMLNGEPIGYAQSYVALGSGDG**R**WEEETDP<br>GVRGID**QL**LANASQLGKGLGTKLVRALVELLFNDPEVTKIQTDPSPSNLRAIRCYEKAGFERQGTVTTP**Y**GPAVYMVQTRQAFERTRSDA*
10	Ib-cr	MSNAKTKLGITKYSIVTNSNDSVTLRLMTEHDLAMLYEWLNRSHIVEWWGGEEARPTLADVQEQYLPSVLAQESVTPYIAMLNGEPIGYAQSYVALGSGDG**R**WEEETDP<br>GVRGID**QL**LANASQLGKGLGTKLVRALVELLFNDPEVTKIQTDPSPSNLRAIRCYEKAGFERQGTVTTP**Y**GPAVYMVQTRQAFERTRSDA*
11	Ib-cr	MSNAKTKLGITKYSIVTNSNDSVTLRLMTEHDLAMLYEWLNRSHIVEWWGGEEARPTLADVQEQYLPSVLAQESVTPYIAMLNGEPIGYAQSYVALGSGDG**R**WEEETDP<br>GVRGID**QL**LANASQLGKGLGTKLVRALVELLFNDPEVTKIQTDPSPSNLRAIRCYEKAGFERQGTVTTP**Y**GPAVYMVQTRQAFERTRSDA*
12	Ia	LNYSICDIAESNELILEAAKMLKKSFLDAGNESWGDIKNAIEEVEECIEHPNICLGICLDDKLIGWTGLRPMYDKTWELHPMVIKTEYQGKGFGKVLLRELETRAKSRG<br>IIGIALGTDDEYQKTSLSMIDINERNIFDEIGNIKNVNNHPYEFYKKCGYMIVGIIPNANGKRKPDIWMWKDIS*
13	Ib$_4$	VTNSNDSVTLRLMTEHDLAMLYEWLNRSHIVEWWGGEEARPTLADVQEQYLPSVLAQESVTPYIAMLNGEPIGYAQSYVALGSGDG**W**WEEETDP<br>GVRGID**QS**LANASQLGKGLGTKLVRALVELLFNDPEVTKIQTDPSPSNLRAIRCYEKAGFERQGTVTTP**D**GPAVYMVQTRQAFERTRSDA*
14	Ib-cr	MSNAKTKLGITKYSIVTNSNDSVTLRLMTEHDLAMLYEWLNRSHIVEWWGGEEARPTLADVQEQYLPSVLAQESVTPYIAMLNGEPIGYAQSYVALGSGDG**R**WEEETDP<br>GVRGID**QL**LANASQLGKGLGTKLVRALVELLFNDPEVTKIQTDPSPSNLRAIRCYEKAGFERQGTVTTP**Y**GPAVYMVQTRQAFERTRSDA*
15	Ib-cr	MSNAKTKLGITKYSIVTNSNDSVTLRLMTEHDLAMLYEWLNRSHIVEWWGGEEARPTLADVQEQYLPSVLAQESVTPYIAMLNGEPIGYAQSYVALGSGDG**R**WEEETDP<br>GVRGID**QL**LANASQLGKGLGTKLVRALVELLFNDPEVTKIQTDPSPSNLRAIRCYEKAGFERQGTVTTP**Y**GPAVYMVQTRQAFERTRSDA*
16	Ib-cr	MSNAKTKLGITKYSIVTNSNDSVTLRLMTEHDLAMLYEWLNRSHIVEWWGGEEARPTLADVQEQYLPSVLAQESVTPYIAMLNGEPIGYAQSYVALGSGDG**R**WEEETDP<br>GVRGID**QL**LANASQLGKGLGTKLVRALVELLFNDPEVTKIQTDPSPSNLRAIRCYEKAGFERQGTVTTP**Y**GPAVYMVQTRQAFERTRSDA*
17	Ib-cr	MSNAKTKLGITKYSIVTNSNDSVTLRLMTEHDLAMLYEWLNRSHIVEWWGGEEARPTLADVQEQYLPSVLAQESVTPYIAMLNGEPIGYAQSYVALGSGDG**R**WEEETDP<br>GVRGID**QL**LANASQLGKGLGTKLVRALVELLFNDPEVTKIQTDPSPSNLRAIRCYEKAGFERQGTVTTP**Y**GPAVYMVQTRQAFERTRSDA
18	Ib$_4$	MTNSNDSVTLRLMTEHDLAMLYEWLNRSHIVEWWGGEEARPTLADVQEQYLPSVLAQESVTPYIAMLNGEPIGYAQSYVALGSGDG**W**WEEETDP<br>GVRGID**QS**LANASQLGKGLGTKLVRALVELLFNDPEVTKIQTDPSPSNLRAIRCYEKAGFEKQGTVTTP**D**GPAVYMVQTRQAFERTRSDA
19	Ib$_4$	MTNSNDSVTLRLMTEHDLAMLYEWLNRSHIVEWWGGEEARPTLADVQEQYLPSVLAQESVTPYIAMLNGEPIGYAQSYVALGSGDG**W**WEEETDP<br>GVRGID**QS**LANASQLGKGLGTKLVRALVELLFNDPEVTKIQTDPSPSNLRAIRCYEKAGFEKQGTVTTP**D**GPAVYMVQTRQAFERTRSDA
20	Ib-cr	MSNAKTKLGITKYSIVTNSNDSVTLRLMTEHDLAMLYEWLNRSHIVEWWGGEEARPTLADVQEQYLPSVLAQESVTPYIAMLNGEPIGYAQSYVALGSGDG**R**WEEETDP<br>GVRGID**QL**LANASQLGKGLGTKLVRALVELLFNDPEVTKIQTDPSPSNLRAIRCYEKAGFERQGTVTTP**Y**GPAVYMVQTRQAFERTRSDA

Amino acid positions 87, 101, 102, and 164 are highlighted in bold (amino acid numbering as per Fig. S1).

Table S3. Gentamicin C MICs (μg/mL) for clinical isolates of *aac(6´)* subtypes I and II

	WT	***E. coli aac(6´)-I***		***E. coli aac(6´)-II***	
		**AG175**	**AG176**	**AG804**	**AG805**
Gentamicin C1	0.5	1	2	4	16
Gentamicin C2	0.5	1	1-2	8	16-32
Gentamicin C1a	0.25-0.5	2-4	2-4	4-8	8-16
Gentamicin C2a	0.5	0.5-1	1	16-32	32-64
Gentamicin C2b	0.5-1	4-8	4-8	8-16	32-64

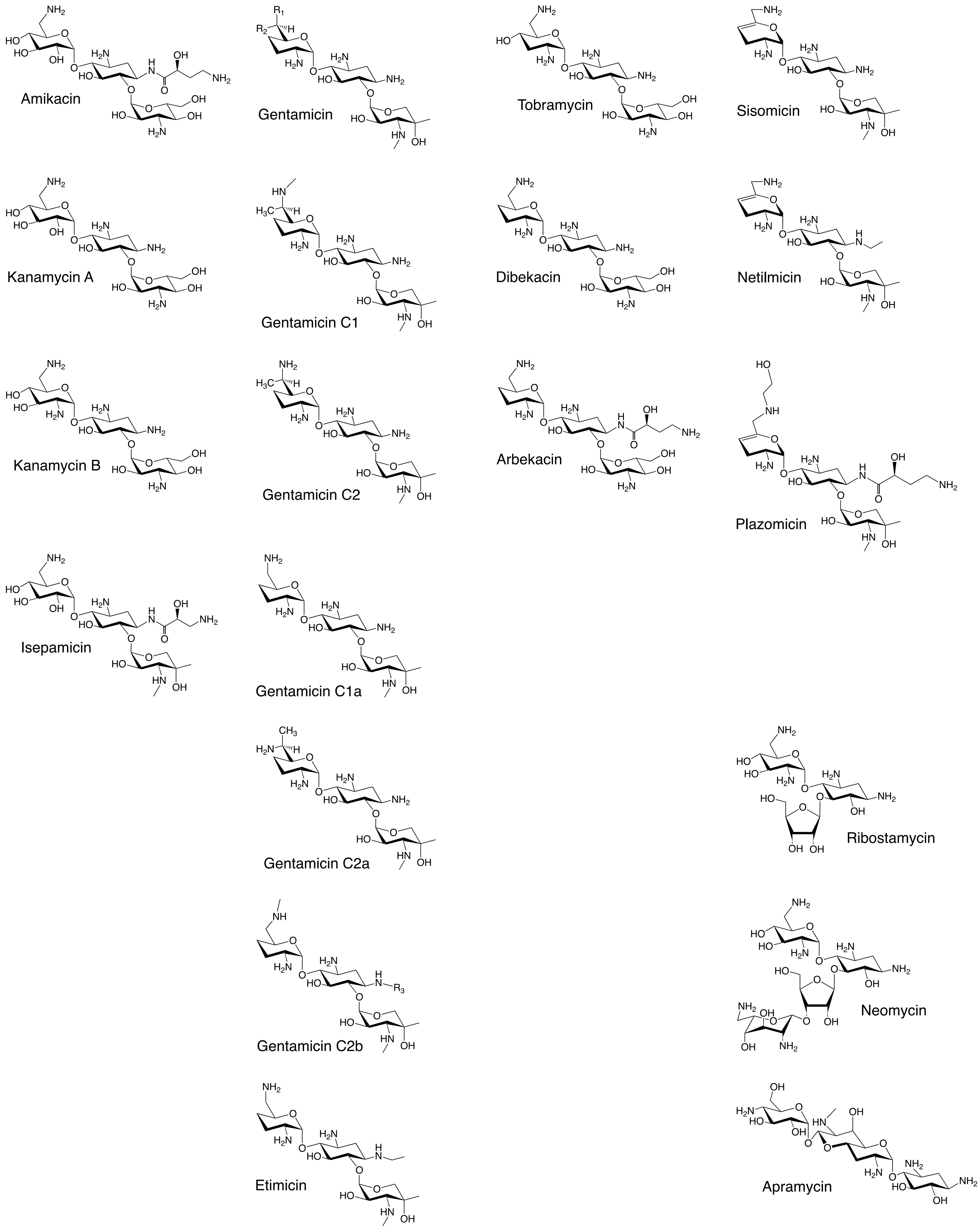

Figure S1. Chemical structures of 2-deoxystreptamine aminoglycosides used in this study.

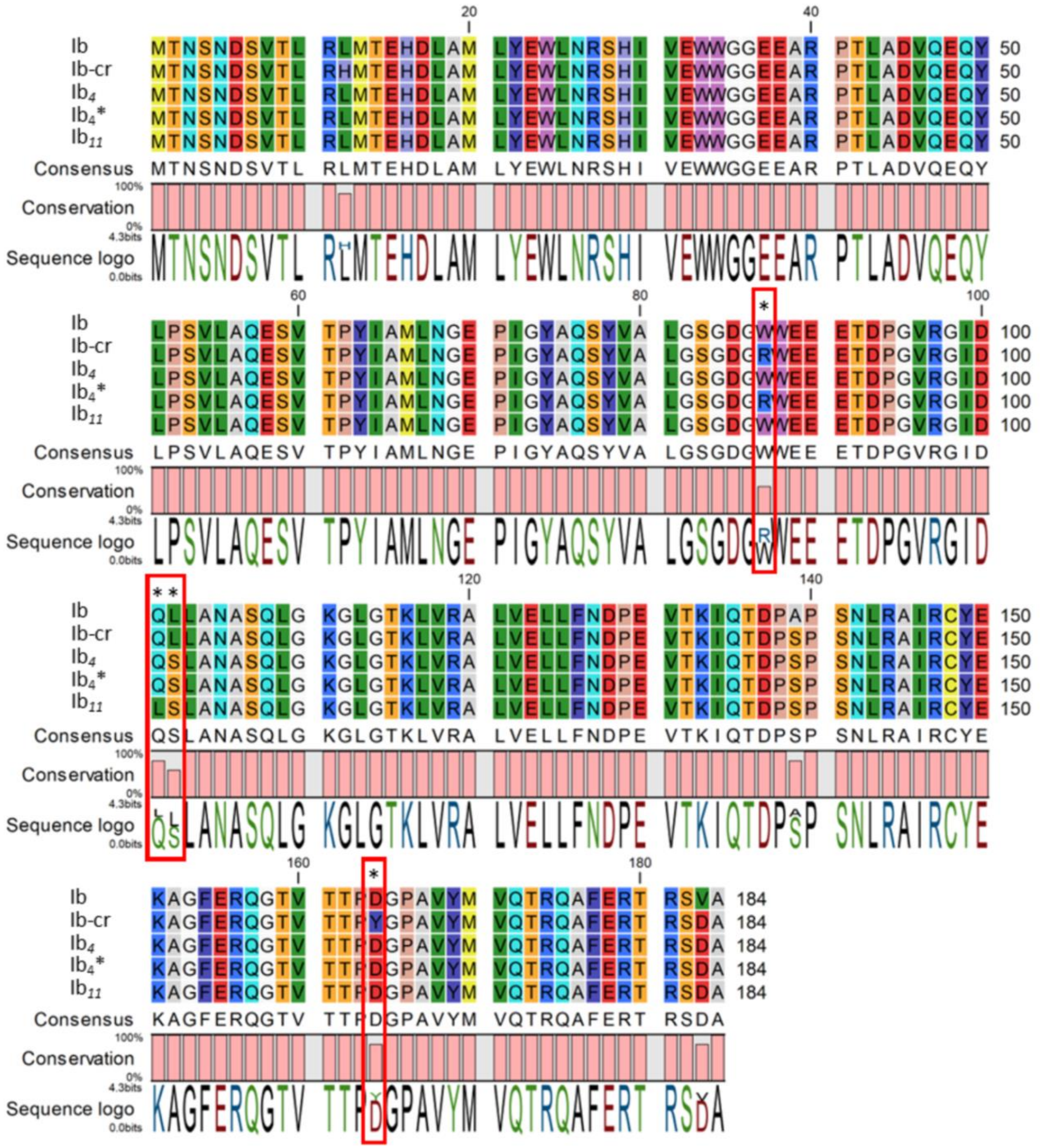

Figure S2. Amino acid sequence alignment of AAC(6′)-Ib variants. The phenotypically relevant mutations are boxed in red. The numbering of amino acids is based on trimmed sequences only showing the core region after removing potential leader sequences. AAC(6′)-Ib-cr is characterized by two mutations Trp87Arg and Asp164Tyr. The latter has also been described as Asp179Tyr, depending on protein length. AAC(6′)-Ib$_4$ is a Leu102Ser variant (also known as Leu119Ser). AAC(6′)-Ib$_{11}$ is characterized by a double substitution Gln101Leu and Leu102Ser (also known as Gln118Leu and Leu119Ser). A new sequence variant of AAC(6′)-Ib$_4$ was discovered that contains the fluoroquinolone-permissive Trp87Arg mutation that has thus far only been described in the context of subtype Ib-cr. This variant (WP_117065919.1) is labeled with an asterisk in the present alignment (AAC(6′)-Ib$_4$*).

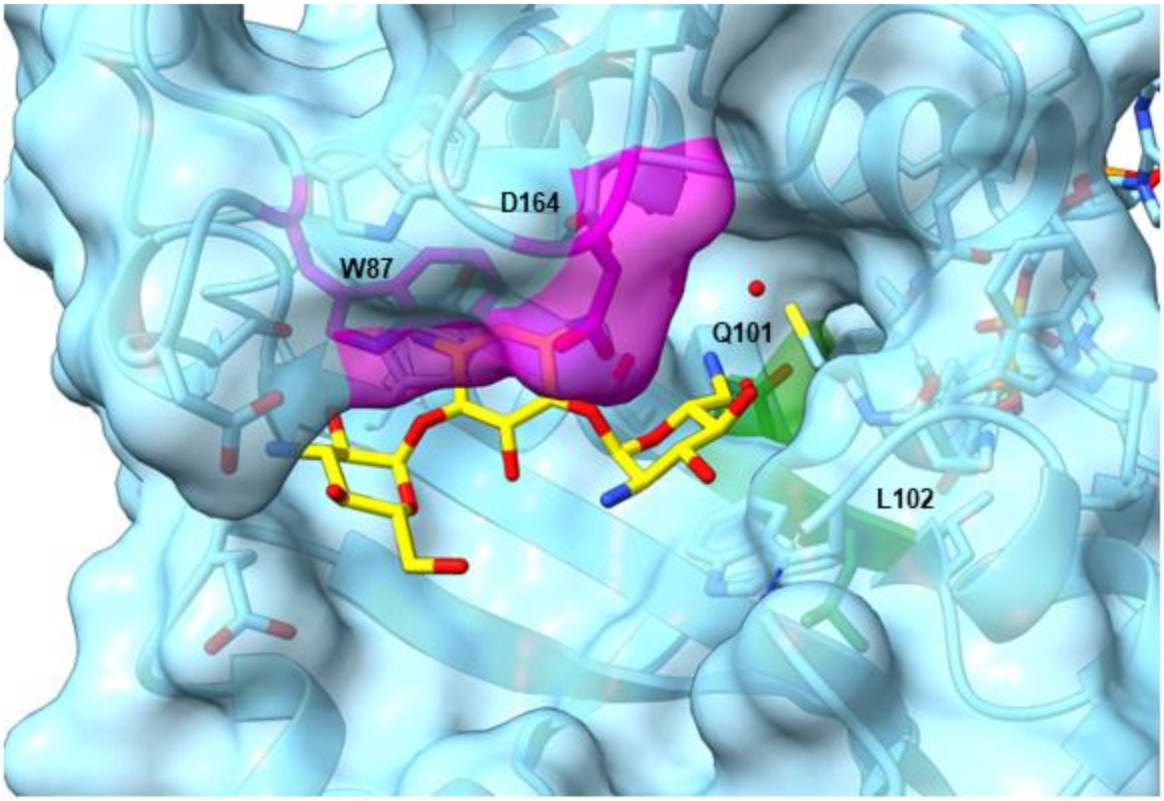

Figure S3. Location of relevant amino acid substitutions in AAC(6′)-Ib variants. Surface structure of AAC(6′)-Ib in complex with kanamycin B (yellow) and co-enzyme A (sky blue, covered) (PDB ID: 2qir) with the four differential amino acid positions 87, 101, 102, and 164 highlighted (amino acid numbering as defined in Figure S1, which is not identical to the numbering in PDB ID 2qir). The clinically most abundant variant AAC(6′)-Ib-cr varies from AAC(6′)-Ib in positions 87 and 164 (in magenta) in the substrate binding pocket facing kanamycin A (yellow sticks) but shares the same aminoglycoside substrate specificity as AAC(6′)-Ib. In contrast, AAC(6′)-Ib_4 and AAC(6′)-Ib_{11} differ from AAC(6′)-Ib in positions 101 and 102 (in green) instead, changing the substrate specificity to result in subtype II and IV phenotypes, respectively. Amino acids Q101 and D101 are facing the surface of the binding pocket for co-enzyme A (sky blue, covered) close to its sulfur moiety. L102S is not located on the protein surface but buried inside the structure. Modification W87R in AAC(6′)-Ib-cr has previously been shown to expand its substrate promiscuity to include fluoroquinolones. In the present study, we described a W87R variation in AAC(6′)-Ib_4 as well, which to our best knowledge has not been reported before.

Table S4. Reference *aac(6′)* gene catalog of the National Database of Antibiotic Resistant Organisms (NDARO), as of 01 July 2024

#Allele	Gene family	Product name	Refseq protein	Refseq nucleotide	Genbank protein	Genbank nucleotide	Curated refseq start
	aac(6')	aminoglycoside 6'-N-acetyltransferase	WP_004874306.1	NG_052265.1	EEQ11435.1	AALD02000008.1	No
	aac(6')	aminoglycoside 6'-N-acetyltransferase	WP_071846239.1	NG_052254.1	BAM45412.1	AB733642.1	No
	aac(6')	aminoglycoside 6'-N-acetyltransferase	WP_029391459.1	NG_052157.1		AJTQ01000183.1	Yes
	aac(6')	aminoglycoside 6'-N-acetyltransferase	WP_019453091.1	NG_052284.1		ALOV01000006.1	Yes
	aac(6')	aminoglycoside 6'-N-acetyltransferase	WP_004937175.1	NG_052389.1	EMF04571.1	AORJ01000065.1	No
	aac(6')	aminoglycoside 6'-N-acetyltransferase	WP_041036791.1	NG_052449.1	BAO35700.1	AP013063.1	No
	aac(6')	aminoglycoside 6'-N-acetyltransferase	WP_023417224.1	NG_052413.1	ESP99889.1	ASHF01000011.1	No
	aac(6')	AAC(6')-Ia family aminoglycoside 6'-N-acetyltransferase	WP_051208285.1	NG_052294.1		AUIH01000073.1	Yes
	aac(6')	aminoglycoside 6'-N-acetyltransferase	WP_021505085.1	NG_052297.1	ERH70569.1	AVSR01000023.1	No
	aac(6')	aminoglycoside 6'-N-acetyltransferase	WP_023529211.1	NG_052445.1	EST37714.1	AWQV01000373.1	No
	aac(6')	aminoglycoside 6'-N-acetyltransferase	WP_042785406.1	NG_052415.1	AIM23676.1	CP003424.1	No
	aac(6')	aminoglycoside 6'-N-acetyltransferase	WP_015379140.1	NG_052263.1	AGE20052.1	CP003959.1	No
	aac(6')	aminoglycoside 6'-N-acetyltransferase	WP_039639671.1	NG_052428.1	AJC61747.1	CP003987.1	No
	aac(6')	aminoglycoside 6'-N-acetyltransferase	WP_044030410.1	NG_052336.1	AIA46829.1	CP005927.1	No
	aac(6')	aminoglycoside 6'-N-acetyltransferase	WP_046928126.1	NG_054688.1	AJT67207.1	CP007699.2	No
	aac(6')	aminoglycoside 6'-N-acetyltransferase	WP_046930149.1	NG_054689.1	AJT69959.1	CP007699.2	No
	aac(6')	aminoglycoside 6'-N-acetyltransferase	WP_047729810.1	NG_052471.1	AKL42171.1	CP011642.1	No
	aac(6')	aminoglycoside 6'-N-acetyltransferase	WP_060706838.1	NG_052209.1	ALE97986.1	CP012685.1	No
	aac(6')	aminoglycoside 6'-N-acetyltransferase	WP_060560340.1	NG_054690.1	ALL39971.1	CP013046.2	No
	aac(6')	aminoglycoside 6'-N-acetyltransferase	WP_047570454.1	NG_052420.1	AUU10860.1	CP026050.1	No
	aac(6')	aminoglycoside 6'-N-acetyltransferase	WP_049646085.1	NG_052198.1	CNE19069.1	CPYJ01000001.1	No
	aac(6')	aminoglycoside 6'-N-acetyltransferase	WP_049677835.1	NG_052199.1	CNH39072.1	CQBM01000001.1	No
	aac(6')	aminoglycoside 6'-N-acetyltransferase	WP_050141558.1	NG_052200.1	CNK13080.1	CQDZ01000001.1	No
	aac(6')	aminoglycoside 6'-N-acetyltransferase	WP_050537864.1	NG_052201.1	CQH02469.1	CTIO01000003.1	No
	aac(6')	aminoglycoside 6'-N-acetyltransferase	WP_003950682.1	NG_052138.1	EFE83821.1	DS999645.1	No
	aac(6')	aminoglycoside 6'-N-acetyltransferase	WP_009067544.1	NG_052139.1	EFL01423.1	GG657742.1	No
	aac(6')	aminoglycoside 6'-N-acetyltransferase	WP_025304332.1	NG_052448.1	CDG14101.1	HG326223.1	No
	aac(6')	aminoglycoside 6'-N-acetyltransferase	WP_033644545.1	NG_052450.1	ETX42903.1	JAPC01000008.1	No
	aac(6')	aminoglycoside 6'-N-acetyltransferase	WP_033636082.1	NG_052451.1	ETX44633.1	JAPD01000005.1	No
	aac(6')	aminoglycoside 6'-N-acetyltransferase	WP_033651281.1	NG_052178.1	EZQ63051.1	JJMZ01000006.1	No
	aac(6')	aminoglycoside 6'-N-acetyltransferase	WP_033639326.1	NG_052346.1	KFD15114.1	JMPQ01000033.1	No
	aac(6')	aminoglycoside 6'-N-acetyltransferase	WP_033268654.1	NG_052341.1		JNZA01000018.1	Yes
	aac(6')	aminoglycoside 6'-N-acetyltransferase	WP_031084431.1	NG_052342.1		JOGT01000014.1	Yes
	aac(6')	aminoglycoside 6'-N-acetyltransferase	WP_030695745.1	NG_054655.1		JOGU01000006.1	Yes
	aac(6')	aminoglycoside 6'-N-acetyltransferase	WP_033654785.1	NG_052345.1	KFB54973.1	JPOB01000020.1	No
	aac(6')	aminoglycoside 6'-N-acetyltransferase	WP_033632034.1	NG_052347.1	KFF87932.1	JPUX01000001.1	No
	aac(6')	aminoglycoside 6'-N-acetyltransferase	WP_046687856.1	NG_052466.1	KKO56015.1	JSFB01000001.1	No
	aac(6')	aminoglycoside 6'-N-acetyltransferase	WP_071846333.1	NG_052419.1	PNO64647.1	JTBC02000011.1	No
	aac(6')	aminoglycoside 6'-N-acetyltransferase	WP_071846334.1	NG_052423.1		JTVI01000034.1	Yes
	aac(6')	aminoglycoside 6'-N-acetyltransferase	WP_049198488.1	NG_052195.1		JUOM01000037.1	Yes
	aac(6')	aminoglycoside 6'-N-acetyltransferase	WP_049241494.1	NG_052196.1		JVCS01000042.1	Yes
	aac(6')	aminoglycoside 6'-N-acetyltransferase	WP_049208356.1	NG_052194.1		JVIF01000032.1	Yes
	aac(6')	aminoglycoside 6'-N-acetyltransferase	WP_049189080.1	NG_052193.1		JVNH01000105.1	Yes
	aac(6')	aminoglycoside 6'-N-acetyltransferase	WP_049204401.1	NG_052192.1		JVXJ01000057.1	Yes
	aac(6')	aminoglycoside 6'-N-acetyltransferase	WP_049212943.1	NG_052191.1		JVYI01000082.1	Yes
	aac(6')	aminoglycoside 6'-N-acetyltransferase	WP_049270433.1	NG_052190.1		JWBL01000029.1	Yes
	aac(6')	aminoglycoside 6'-N-acetyltransferase	WP_039569351.1	NG_052427.1	KHO40870.1	JWLO01000007.1	No

Table S4 (continued). Reference *aac(6′)* gene catalog of the National Database of Antibiotic Resistant Organisms (NDARO), as of 01 July 2024

#Allele	Gene family	Product name	Refseq protein	Refseq nucleotide	Genbank protein	Genbank nucleotide	Curated refseq start
	aac(6')	aminoglycoside 6'-N-acetyltransferase	WP_071846352.1	NG_052459.1	KIX79344.1	JXOM01000108.1	No
	aac(6')	aminoglycoside 6'-N-acetyltransferase	WP_043911783.1	NG_052429.1	KIQ65506.1	JXZB01000002.1	No
	aac(6')	aminoglycoside 6'-N-acetyltransferase	WP_026328431.1	NG_052533.1		KB905816.1	Yes
	aac(6')	aminoglycoside 6'-N-acetyltransferase	WP_019655053.1	NG_052534.1		KB907453.1	Yes
	aac(6')	aminoglycoside 6'-N-acetyltransferase	WP_038879528.1	NG_052292.1		KE375664.1	Yes
	aac(6')	aminoglycoside 6'-N-acetyltransferase	WP_071846259.1	NG_052300.1	AGY30798.1	KF525275.1	No
	aac(6')	aminoglycoside 6'-N-acetyltransferase	WP_071846260.1	NG_052301.1	AGY30800.1	KF525276.1	No
	aac(6')	aminoglycoside 6'-N-acetyltransferase	WP_071846204.1	NG_052175.1	AHN60086.1	KJ138219.1	No
	aac(6')	aminoglycoside 6'-N-acetyltransferase	WP_047467383.1	NG_052425.1		KN549149.1	Yes
	aac(6')	aminoglycoside 6'-N-acetyltransferase	WP_046502149.1	NG_052464.1		LATD01000220.1	Yes
	aac(6')	aminoglycoside 6'-N-acetyltransferase	WP_047139664.1	NG_052469.1	KLJ03806.1	LAYX01000007.1	No
	aac(6')	aminoglycoside 6'-N-acetyltransferase	WP_046897272.1	NG_052467.1	KKZ18771.1	LCWI01000005.1	No
	aac(6')	aminoglycoside 6'-N-acetyltransferase	WP_047026538.1	NG_052468.1	KLE36360.1	LDEG01000018.1	No
	aac(6')	aminoglycoside 6'-N-acetyltransferase	WP_053518652.1	NG_052204.1	KOQ68998.1	LDVR01000042.1	No
	aac(6')	aminoglycoside 6'-N-acetyltransferase	WP_048234935.1	NG_052472.1	KLX11493.1	LETV01000019.1	No
	aac(6')	aminoglycoside 6'-N-acetyltransferase	WP_048325528.1	NG_052187.1	KMJ12223.1	LFBS01000007.1	No
	aac(6')	aminoglycoside 6'-N-acetyltransferase	WP_049443010.1	NG_052189.1	KMU66930.1	LFKU01000005.1	No
	aac(6')	aminoglycoside 6'-N-acetyltransferase	WP_030403494.1	NG_052205.1	KOU01101.1	LGCX01000149.1	No
	aac(6')	aminoglycoside 6'-N-acetyltransferase	WP_053638058.1	NG_052206.1	KOX34026.1	LGEF01000079.1	No
	aac(6')	aminoglycoside 6'-N-acetyltransferase	WP_049031504.1	NG_052221.1	KVS21672.1	LPCS01000005.1	No
aac(6')-32	aac(6')	aminoglycoside N-acetyltransferase AAC(6')-32	WP_032491968.1	NG_047266.1	ABR10839.1	EF614235.1	No
aac(6')-33	aac(6')	aminoglycoside 6'-N-acetyltransferase AAC(6')-33	WP_015059044.1	NG_047267.1	ACT99625.1	GQ337064.1	No
aac(6')-I30	aac(6')	aminoglycoside 6'-N-acetyltransferase AAC(6')-I30	WP_063840274.1	NG_047268.1	AAP43642.1	AY289608.1	No
aac(6')-Iae	aac(6')	aminoglycoside 6'-N-acetyltransferase AAC(6')-Iae	WP_003159545.1	NG_047277.1	BAD14386.1	AB104852.1	No
aac(6')-Iaf	aac(6')	aminoglycoside 6'-N-acetyltransferase AAC(6')-Iaf	WP_063840278.1	NG_047278.1	BAH66386.1	AB462903.1	No
aac(6')-Iai	aac(6')	aminoglycoside 6'-N-acetyltransferase AAC(6')-Iai	WP_063840279.1	NG_047279.1	ACI28880.1	EU886977.1	No
aac(6')-Iaj	aac(6')	aminoglycoside 6'-N-acetyltransferase AAC(6')-Iaj	WP_069174568.1	NG_051491.1	BAM46120.1	AB709942.1	No
aac(6')-Ial	aac(6')	aminoglycoside N-acetyltransferase AAC(6')-Ial	WP_016930164.1	NG_047281.1	BAO05514.1	AB871481.1	No
aac(6')-Ic	aac(6')	aminoglycoside N-acetyltransferase AAC(6')-Ic	WP_033649026.1	NG_047294.1	AAA26549.1	M94066.1	No
aac(6')-Isa	aac(6')	aminoglycoside N-acetyltransferase AAC(6')-Isa	WP_016575755.1	NG_047311.1	BAD10948.2	AB116646.1	No
aacA16	aac(6')	AAC(6')-Ia family aminoglycoside 6'-N-acetyltransferase AacA16	WP_001109644.1	NG_052380.1	ADX02779.1	CP001921.1	No
aacA47	aac(6')	aminoglycoside 6'-N-acetyltransferase AacA47	WP_041550454.1	NG_052259.1		CP003601.1	Yes
aacA56	aac(6')	AAC(6')-Ia family aminoglycoside 6'-N-acetyltransferase AacA56	WP_045890872.1	NG_051492.1	AIT97187.1	KM201605.1	No
	aac(6')-29	aminoglycoside 6'-N-acetyltransferase AAC(6')-29	WP_039110076.1	NG_052220.1	KVJ93461.1	LRJS01000061.1	No
aac(6')-29a	aac(6')-29	aminoglycoside N-acetyltransferase AAC(6')-29a	WP_064190968.1	NG_048575.1		AF263519.1	Yes
aac(6')-29b	aac(6')-29	aminoglycoside N-acetyltransferase AAC(6')-29b	WP_064190969.1	NG_048576.1		AF263519.1	Yes
	aac(6')-30	aminoglycoside 6'-N-acetyltransferase AAC(6')-30	WP_051645529.1	NG_051559.1	KDF29949.1	JMUP01000027.1	No
	aac(6')-30/ aac(6')-Ib'	bifunctional aminoglycoside N-acetyltransferase AAC(6')-30/aminoglycoside N-acetyltransferase AAC(6')-Ib'	WP_063838853.1	NG_047213.1	CAE48335.2	AJ584652.2	No
	aac(6')-31	aminoglycoside N-acetyltransferase AAC(6')-31	WP_044424439.1	NG_047265.1	CAK55563.1	AM283490.1	No
	aac(6')-31	aminoglycoside N-acetyltransferase AAC(6')-31	WP_054914207.1	NG_052210.1		BCBD01000273.1	Yes
	aac(6')-31	aminoglycoside N-acetyltransferase AAC(6')-31	WP_058124867.1	NG_052214.1	ALO40181.1	CP013119.1	No
	aac(6')-35	aminoglycoside 6'-N-acetyltransferase AAC(6')-35	WP_087349651.1	NG_063816.1	ATL63228.1	KY753879.1	No
	aac(6')-I	AAC(6')-Ighjkrstuvwx family aminoglycoside N-acetyltransferase	WP_008940904.1	NG_052523.1	EEH69174.1	ABYN01000078.1	No
	aac(6')-I	AAC(6')-Ighjkrstuvwx family aminoglycoside N-acetyltransferase	WP_017395772.1	NG_052255.1		AMJB01000211.1	Yes
	aac(6')-I	AAC(6')-Ighjkrstuvwx family aminoglycoside N-acetyltransferase	WP_009510932.1	NG_052258.1	EKU56564.1	AMZS01000035.1	No
	aac(6')-I	AAC(6')-Ighjkrstuvwx family aminoglycoside N-acetyltransferase	WP_004670361.1	NG_052394.1	ENU30617.1	APOK01000028.1	No

Table S4 (continued). Reference *aac(6′)* gene catalog of the National Database of Antibiotic Resistant Organisms (NDARO), as of 01 July 2024

#Allele	Gene family	Product name	Refseq protein	Refseq nucleotide	Genbank protein	Genbank nucleotide	Curated refseq start
	aac(6')-I	AAC(6')-Ighjkrstuvwx family aminoglycoside N-acetyltransferase	WP_004770322.1	NG_052395.1	ENU94191.1	APPC01000001.1	No
	aac(6')-I	AAC(6')-Ighjkrstuvwx family aminoglycoside N-acetyltransferase	WP_004801433.1	NG_052396.1	ENV11327.1	APPH01000003.1	No
	aac(6')-I	AAC(6')-Ighjkrstuvwx family aminoglycoside N-acetyltransferase	WP_005062866.1	NG_052397.1	ENW03190.1	APQL01000012.1	No
	aac(6')-I	AAC(6')-Ighjkrstuvwx family aminoglycoside N-acetyltransferase	WP_005091170.1	NG_052398.1	ENW19086.1	APQR01000012.1	No
	aac(6')-I	AAC(6')-Ighjkrstuvwx family aminoglycoside N-acetyltransferase	WP_005145021.1	NG_052399.1	ENW78703.1	APRH01000028.1	No
	aac(6')-I	AAC(6')-Ighjkrstuvwx family aminoglycoside N-acetyltransferase	WP_005184197.1	NG_052401.1	ENW95138.1	APRL01000003.1	No
	aac(6')-I	AAC(6')-Ighjkrstuvwx family aminoglycoside N-acetyltransferase	WP_005197871.1	NG_052400.1	ENW94573.1	APRM01000012.1	No
	aac(6')-I	AAC(6')-Ighjkrstuvwx family aminoglycoside N-acetyltransferase	WP_005202238.1	NG_052529.1	ENX58828.1	APRN01000035.1	No
	aac(6')-I	AAC(6')-Ighjkrstuvwx family aminoglycoside N-acetyltransferase	WP_005243483.1	NG_052525.1	ENX12286.1	APRT01000022.1	No
	aac(6')-I	AAC(6')-Ighjkrstuvwx family aminoglycoside N-acetyltransferase	WP_005259042.1	NG_052526.1	ENX20722.1	APRW01000012.1	No
	aac(6')-I	AAC(6')-Ighjkrstuvwx family aminoglycoside N-acetyltransferase	WP_005269797.1	NG_052527.1	ENX36275.1	APRZ01000006.1	No
	aac(6')-I	AAC(6')-Ighjkrstuvwx family aminoglycoside N-acetyltransferase	WP_005288246.1	NG_052528.1	ENX36670.1	APSA01000010.1	No
	aac(6')-I	AAC(6')-Ighjkrstuvwx family aminoglycoside N-acetyltransferase	WP_016162901.1	NG_052166.1	EOR09207.1	AQFL01000007.1	No
	aac(6')-I	AAC(6')-Ighjkrstuvwx family aminoglycoside N-acetyltransferase	WP_032874497.1	NG_052457.1	EXB26010.1	JEWN01000004.1	No
	aac(6')-I	AAC(6')-Ighjkrstuvwx family aminoglycoside N-acetyltransferase	WP_032870384.1	NG_052458.1	EXB46212.1	JEWS01000006.1	No
	aac(6')-I	AAC(6')-Ighjkrstuvwx family aminoglycoside N-acetyltransferase	WP_047426753.1	NG_052421.1	KHF78826.1	JSZD01000004.1	No
	aac(6')-I	AAC(6')-Ighjkrstuvwx family aminoglycoside N-acetyltransferase	WP_032880374.1	NG_052531.1		KB850109.1	Yes
	aac(6')-I	AAC(6')-Ighjkrstuvwx family aminoglycoside N-acetyltransferase	WP_071846216.1	NG_052216.1	ALS88215.1	KT778788.1	No
	aac(6')-I	aminoglycoside 6'-N-acetyltransferase	WP_002289795.1	NG_052371.1	EAN08580.1	AAAK03000130.1	No
	aac(6')-I	aminoglycoside 6'-N-acetyltransferase	WP_002293569.1	NG_052275.1	EFF36162.1	ABQA01000128.1	No
	aac(6')-I	aminoglycoside 6'-N-acetyltransferase	WP_002293989.1	NG_052140.1	EEI59381.1	ACHL01000139.1	No
	aac(6')-I	aminoglycoside 6'-N-acetyltransferase	WP_019722502.1	NG_052504.1		AFWZ01000007.1	Yes
	aac(6')-I	aminoglycoside 6'-N-acetyltransferase	WP_002328174.1	NG_052260.1	ELA64846.1	AHWN01000015.1	No
	aac(6')-I	aminoglycoside 6'-N-acetyltransferase	WP_002339746.1	NG_052261.1	ELB23361.1	AHXK01000039.1	No
	aac(6')-I	aminoglycoside 6'-N-acetyltransferase	WP_016172918.1	NG_052169.1	EOT40837.1	AHYR01000006.1	No
	aac(6')-I	aminoglycoside 6'-N-acetyltransferase	WP_016177698.1	NG_052168.1	EOT29546.1	AHYU01000052.1	No
	aac(6')-I	aminoglycoside 6'-N-acetyltransferase	WP_010723742.1	NG_052161.1	EOH47286.1	AITZ01000012.1	No
	aac(6')-I	aminoglycoside 6'-N-acetyltransferase	WP_010727362.1	NG_052159.1	EOG05128.1	AIUW01000009.1	No
	aac(6')-I	aminoglycoside 6'-N-acetyltransferase	WP_010728104.1	NG_052160.1	EOG13098.1	AIVB01000005.1	No
	aac(6')-I	aminoglycoside 6'-N-acetyltransferase	WP_010736157.1	NG_052162.1	EOH60829.1	AJAH01000025.1	No
	aac(6')-I	aminoglycoside 6'-N-acetyltransferase	WP_010750427.1	NG_052164.1	EOH92539.1	AJAN01000013.1	No
	aac(6')-I	aminoglycoside 6'-N-acetyltransferase	WP_010752789.1	NG_052163.1	EOH90388.1	AJAP01000004.1	No
	aac(6')-I	aminoglycoside 6'-N-acetyltransferase	WP_010768325.1	NG_052165.1	EOL44032.1	AJAT01000014.1	No
	aac(6')-I	aminoglycoside 6'-N-acetyltransferase	WP_002373947.1	NG_052285.1	EJY45308.1	AMBL01000051.1	No
	aac(6')-I	aminoglycoside 6'-N-acetyltransferase	WP_023519215.1	NG_052446.1	BAO06172.1	AP013036.1	No
	aac(6')-I	aminoglycoside 6'-N-acetyltransferase	WP_016628216.1	NG_052290.1	EPI08854.1	ATIS01000069.1	No
	aac(6')-I	aminoglycoside 6'-N-acetyltransferase	WP_024635824.1	NG_052447.1	KEI48560.1	AWWN01000104.1	No
	aac(6')-I	aminoglycoside 6'-N-acetyltransferase	WP_010737153.1	NG_052281.1	AFM70383.1	CP003504.1	No
	aac(6')-I	aminoglycoside 6'-N-acetyltransferase	WP_048603867.1	NG_052188.1		CVRN01000006.1	Yes
	aac(6')-I	aminoglycoside 6'-N-acetyltransferase	WP_034688908.1	NG_052177.1	EYT95687.1	JDFT01000009.1	No
	aac(6')-I	aminoglycoside 6'-N-acetyltransferase	WP_034700626.1	NG_052337.1	KDR91621.1	JMIG01000016.1	No
	aac(6')-I	aminoglycoside 6'-N-acetyltransferase	WP_034860925.1	NG_052291.1		KE351596.1	Yes
	aac(6')-I	aminoglycoside 6'-N-acetyltransferase	WP_071846353.1	NG_052465.1	KKJ73856.1	LBIL01000048.1	No
	aac(6')-I	aminoglycoside 6'-N-acetyltransferase	WP_053766315.1	NG_052207.1		LGAO01000043.1	Yes
aac(6')-Ig	aac(6')-I	aminoglycoside N-acetyltransferase AAC(6')-Ig	WP_005081764.1	NG_047296.1	AAA21889.1	L09246.1	No
aac(6')-Ih	aac(6')-I	aminoglycoside N-acetyltransferase AAC(6')-Ih	WP_016541245.1	NG_047297.1	AAC41391.1	L29044.1	No
aac(6')-Ii	aac(6')-I	aminoglycoside N-acetyltransferase AAC(6')-Ii	WP_008265821.1	NG_047298.1	AAB63533.1	L12710.1	No

Table S4 (continued). Reference *aac(6′)* gene catalog of the National Database of Antibiotic Resistant Organisms (NDARO), as of 01 July 2024

#Allele	Gene family	Product name	Refseq protein	Refseq nucleotide	Genbank protein	Genbank nucleotide	Curated refseq start
aac(6')-Iid	aac(6')-I	aminoglycoside N-acetyltransferase AAC(6')-Iid	WP_010720790.1	NG_047299.1	CAE50925.1	AJ584700.2	No
aac(6')-Iih	aac(6')-I	aminoglycoside N-acetyltransferase AAC(6')-Iih	WP_005880220.1	NG_047300.1	CAE50926.1	AJ584701.2	No
aac(6')-Ij	aac(6')-I	aminoglycoside N-acetyltransferase AAC(6')-Ij	WP_016651650.1	NG_047301.1	AAC41392.1	L29045.1	No
aac(6')-Ik	aac(6')-I	aminoglycoside N-acetyltransferase AAC(6')-Ik	WP_063840323.1	NG_047302.1	AAA87229.1	L29510.1	No
aac(6')-Ir	aac(6')-I	aminoglycoside N-acetyltransferase AAC(6')-Ir	WP_063840327.1	NG_047309.1	AAD03490.1	AF031326.1	No
aac(6')-Is	aac(6')-I	aminoglycoside N-acetyltransferase AAC(6')-Is	WP_063840328.1	NG_047310.1	AAD03491.1	AF031327.1	No
aac(6')-It	aac(6')-I	aminoglycoside N-acetyltransferase AAC(6')-It	WP_063840329.1	NG_047312.1	AAD03492.1	AF031328.1	No
aac(6')-Iu	aac(6')-I	aminoglycoside N-acetyltransferase AAC(6')-Iu	WP_005208202.1	NG_047313.1	AAD03493.1	AF031329.1	No
aac(6')-Iv	aac(6')-I	aminoglycoside N-acetyltransferase AAC(6')-Iv	WP_004652049.1	NG_047314.1	AAD03494.1	AF031330.1	No
aac(6')-Iw	aac(6')-I	aminoglycoside N-acetyltransferase AAC(6')-Iw	WP_005296085.1	NG_047315.1	AAD03495.1	AF031331.1	No
aac(6')-Ix	aac(6')-I	aminoglycoside N-acetyltransferase AAC(6')-Ix	WP_063840330.1	NG_047316.1	AAD03496.1	AF031332.1	No
aac(6')-IIb	aac(6')-II	aminoglycoside N-acetyltransferase AAC(6')-IIb	WP_063840276.1	NG_047272.1	AAA25680.1	L06163.1	No
aacA35	aac(6')-II	AAC(6')-II family aminoglycoside 6'-N-acetyltransferase AacA35	WP_024437054.1	NG_047269.1	CAH19071.1	AJ628983.2	No
	aac(6')-III	tobramycin N-acetyltransferase AAC(6')-III	WP_010107623.1	NG_068500.1	AIO69677.1	CP008727.1	No
	aac(6')-III	tobramycin N-acetyltransferase AAC(6')-III	WP_038790798.1	NG_068501.1	AIV74302.1	CP009156.1	No
	aac(6')-III	tobramycin N-acetyltransferase AAC(6')-III	WP_038726674.1	NG_068502.1	APY96494.1	CP017045.1	No
	aac(6')-IIa	aminoglycoside N-acetyltransferase AAC(6')-IIa	WP_071846189.1	NG_052142.1	ACO53359.1	FJ817422.1	No
	aac(6')-IIa	aminoglycoside N-acetyltransferase AAC(6')-IIa	WP_071846374.1	NG_052505.1	AER57901.1	JN118546.1	No
	aac(6')-IIa	aminoglycoside N-acetyltransferase AAC(6')-IIa	WP_023622803.1	NG_047270.1	AAA25688.1	M29695.1	No
	aac(6')-IIc	aminoglycoside N-acetyltransferase AAC(6')-IIc	WP_012695484.1	NG_047273.1	AAD46626.1	AF162771.1	No
	aac(6')-IIc	aminoglycoside N-acetyltransferase AAC(6')-IIc	WP_045332676.1	NG_052462.1	KJO93645.1	LAAL01000059.1	No
	aac(6')-Ia	aminoglycoside 6'-N-acetyltransferase AAC(6')-Ia	WP_032489895.1	NG_047275.1	AAG45714.1	AF205943.1	No
	aac(6')-Ia	aminoglycoside 6'-N-acetyltransferase AAC(6')-Ia	WP_058677876.1	NG_052217.1	KTK23273.1	LPQV01000084.1	No
	aac(6')-Ia	aminoglycoside 6'-N-acetyltransferase AAC(6')-Ia	WP_013136949.1	NG_051679.1	AAA72107.1	M86913.1	No
	aac(6')-Iad	aminoglycoside N-acetyltransferase AAC(6')-Iad	WP_063840277.1	NG_047276.1	BAD12078.1	AB119105.1	No
	aac(6')-Iak	aminoglycoside N-acetyltransferase AAC(6')-Iak	WP_057493474.1	NG_047280.1	BAO21229.1	AB894482.1	No
	aac(6')-Iak	aminoglycoside N-acetyltransferase AAC(6')-Iak	WP_005418370.1	NG_052391.1	EMI48985.1	APIT01000070.1	No
	aac(6')-Iak	aminoglycoside N-acetyltransferase AAC(6')-Iak	WP_049468785.1	NG_052197.1		JVZQ01000186.1	Yes
	aac(6')-Iak	aminoglycoside N-acetyltransferase AAC(6')-Iak	WP_053461075.1	NG_052203.1	KOO82158.1	JZIW01000001.1	No
	aac(6')-Iak	aminoglycoside N-acetyltransferase AAC(6')-Iak	WP_064239013.1	NG_052202.1	KOO79896.1	JZTX01000001.1	No
	aac(6')-Ian	aminoglycoside N-acetyltransferase AAC(6')-Ian	WP_000960976.1	NG_047282.1	BAQ22025.1	AP014611.1	No
	aac(6')-Ian	aminoglycoside N-acetyltransferase AAC(6')-Ian	WP_051139090.1	NG_052170.1		ANNO01000478.1	Yes
	aac(6')-Ib	AAC(6')-Ib family aminoglycoside 6'-N-acetyltransferase	WP_071846254.1	NG_052283.1	AFP97029.1	JX131372.1	No
	aac(6')-Ib	AAC(6')-Ib family aminoglycoside 6'-N-acetyltransferase	WP_065187201.1	NG_052219.1		LRJJ01000092.1	Yes
	aac(6')-Ib	AAC(6')-Ib family aminoglycoside 6'-N-acetyltransferase	WP_063840283.1	NG_047286.1		AF360376.1	Yes
	aac(6')-Ib	AAC(6')-Ib family aminoglycoside 6'-N-acetyltransferase	WP_063978482.1	NG_052057.1	CAA11472.1	AJ223604.1	No
	aac(6')-Ib	AAC(6')-Ib family aminoglycoside 6'-N-acetyltransferase	WP_063840319.1	NG_047289.1		AY648351.1	Yes
	aac(6')-Ib	AAC(6')-Ib family aminoglycoside 6'-N-acetyltransferase	WP_071846332.1	NG_052418.1		CCHO01000225.1	Yes
	aac(6')-Ib	AAC(6')-Ib family aminoglycoside 6'-N-acetyltransferase	WP_101516660.1	NG_067964.1	AYF71935.1	CP032569.2	No
	aac(6')-Ib	AAC(6')-Ib family aminoglycoside 6'-N-acetyltransferase	WP_085844237.1	NG_067951.1		FXQR01000071.1	Yes
	aac(6')-Ib	AAC(6')-Ib family aminoglycoside 6'-N-acetyltransferase	WP_085843736.1	NG_067950.1		FXRF01000050.1	Yes
	aac(6')-Ib	AAC(6')-Ib family aminoglycoside 6'-N-acetyltransferase	WP_063612062.1	NG_052358.1	ADK11266.1	HM175873.1	No
	aac(6')-Ib	AAC(6')-Ib family aminoglycoside 6'-N-acetyltransferase	WP_071846208.1	NG_052181.1		JMXB01000033.1	Yes
	aac(6')-Ib	AAC(6')-Ib family aminoglycoside 6'-N-acetyltransferase	WP_071846209.1	NG_052182.1		JMYR01000034.1	Yes
	aac(6')-Ib	AAC(6')-Ib family aminoglycoside 6'-N-acetyltransferase	WP_071846373.1	NG_052503.1	AER27705.1	JN091097.1	No
	aac(6')-Ib	AAC(6')-Ib family aminoglycoside 6'-N-acetyltransferase	WP_071846241.1	NG_052262.1	AGC39277.1	JX982232.1	No
	aac(6')-Ib	AAC(6')-Ib family aminoglycoside 6'-N-acetyltransferase	WP_064765190.1	NG_067946.1		KI973262.1	Yes

Table S4 (continued). Reference *aac(6′)* gene catalog of the National Database of Antibiotic Resistant Organisms (NDARO), as of 01 July 2024

#Allele	Gene family	Product name	Refseq protein	Refseq nucleotide	Genbank protein	Genbank nucleotide	Curated refseq start
	aac(6')-Ib	AAC(6')-Ib family aminoglycoside 6'-N-acetyltransferase	WP_071593230.1	NG_052059.1	AIU93991.1	KJ631731.1	No
	aac(6')-Ib	AAC(6')-Ib family aminoglycoside 6'-N-acetyltransferase	WP_063840280.1	NG_056043.1	KSL18989.2	LLQT01000027.1	No
	aac(6')-Ib	AAC(6')-Ib family aminoglycoside 6'-N-acetyltransferase	WP_069067473.1	NG_067948.1	ODH25762.1	LXFA01000149.1	No
	aac(6')-Ib	AAC(6')-Ib family aminoglycoside 6'-N-acetyltransferase	WP_063840281.1	NG_047284.1		M23634.1	Yes
	aac(6')-Ib	AAC(6')-Ib family aminoglycoside 6'-N-acetyltransferase	WP_159287722.1	NG_067957.1	AXN76423.1	MH491967.2	No
	aac(6')-Ib	AAC(6')-Ib family aminoglycoside 6'-N-acetyltransferase	WP_113613509.1	NG_067953.1	RBM71843.1	PYWE01000037.1	No
	aac(6')-Ib	AAC(6')-Ib family aminoglycoside 6'-N-acetyltransferase	WP_123085936.1	NG_067958.1	RNF67660.1	RJAH01000044.1	No
	aac(6')-Ib	AAC(6')-Ib family aminoglycoside 6'-N-acetyltransferase	WP_079452738.1	NG_067961.1	RTU47553.1	RXUW01000169.1	No
	aac(6')-Ib	AAC(6')-Ib family aminoglycoside 6'-N-acetyltransferase	WP_132629969.1	NG_067962.1		SDAC01000257.1	Yes
	aac(6')-Ib	AAC(6')-Ib family aminoglycoside 6'-N-acetyltransferase	WP_117046752.1	NG_067954.1		UKPL01000058.1	Yes
	aac(6')-Ib	AAC(6')-Ib family aminoglycoside 6'-N-acetyltransferase	WP_140423191.1	NG_065404.1		HE653230.1	Yes
	aac(6')-Ib	AAC(6')-Ib family aminoglycoside 6'-N-acetyltransferase	WP_117065919.1	NG_067955.1		UKRD01000041.1	Yes
	aac(6')-Ib	AAC(6')-Ib family aminoglycoside 6'-N-acetyltransferase	WP_071846301.1	NG_052361.1	BAD73861.1	AB195796.1	No
	aac(6')-Ib	AAC(6')-Ib family aminoglycoside 6'-N-acetyltransferase	WP_071593232.1	NG_052063.1	AAK49459.1	AF315786.1	No
	aac(6')-Ib	AAC(6')-Ib family aminoglycoside 6'-N-acetyltransferase	WP_022631162.1	NG_052535.1	ERN61539.1	AVAN01000004.1	No
	aac(6')-Ib	AAC(6')-Ib family aminoglycoside 6'-N-acetyltransferase	WP_013250882.1	NG_047288.1	AAO32357.1	AY219651.1	No
	aac(6')-Ib	AAC(6')-Ib family aminoglycoside 6'-N-acetyltransferase	WP_134628915.1	NG_067963.1		CAADQB010001296.1	Yes
	aac(6')-Ib	AAC(6')-Ib family aminoglycoside 6'-N-acetyltransferase	WP_071984682.1	NG_067949.1		CP017073.1	Yes
	aac(6')-Ib	AAC(6')-Ib family aminoglycoside 6'-N-acetyltransferase	WP_104442471.1	NG_067952.1	AUX88330.1	CP026418.1	No
	aac(6')-Ib	AAC(6')-Ib family aminoglycoside 6'-N-acetyltransferase	WP_151405943.1	NG_067965.1	QDE47050.1	CP041052.1	No
	aac(6')-Ib	AAC(6')-Ib family aminoglycoside 6'-N-acetyltransferase	WP_071846361.1	NG_052481.1	ABN13423.2	EF368053.1	No
	aac(6')-Ib	AAC(6')-Ib family aminoglycoside 6'-N-acetyltransferase	WP_071846382.1	NG_052517.1	ACA23032.1	EU434618.1	No
	aac(6')-Ib	AAC(6')-Ib family aminoglycoside 6'-N-acetyltransferase	WP_071846385.1	NG_052520.1	ACH68560.1	FJ157994.1	No
	aac(6')-Ib	AAC(6')-Ib family aminoglycoside 6'-N-acetyltransferase	WP_071846249.1	NG_052274.1		FN554982.1	Yes
	aac(6')-Ib	AAC(6')-Ib family aminoglycoside 6'-N-acetyltransferase	WP_071846291.1	NG_052349.1		FN824509.1	Yes
	aac(6')-Ib	AAC(6')-Ib family aminoglycoside 6'-N-acetyltransferase	WP_022631510.1	NG_047291.1	AFJ11384.1	JQ808129.1	No
	aac(6')-Ib	AAC(6')-Ib family aminoglycoside 6'-N-acetyltransferase	WP_071846329.1	NG_052414.1	AHA61211.1	KF040452.1	No
	aac(6')-Ib	AAC(6')-Ib family aminoglycoside 6'-N-acetyltransferase	WP_071593200.1	NG_052024.1	AGY30801.1	KF525277.1	No
	aac(6')-Ib	AAC(6')-Ib family aminoglycoside 6'-N-acetyltransferase	WP_071846262.1	NG_052303.1	AGY30802.1	KF525278.1	No
	aac(6')-Ib	AAC(6')-Ib family aminoglycoside 6'-N-acetyltransferase	WP_071846281.1	NG_052328.1	AIA09165.1	KJ561897.1	No
	aac(6')-Ib	AAC(6')-Ib family aminoglycoside 6'-N-acetyltransferase	WP_012695458.1	NG_051697.1	AAW29412.1	L06822.4	No
	aac(6')-Ib	AAC(6')-Ib family aminoglycoside 6'-N-acetyltransferase	WP_072201103.1	NG_067947.1		LPQV01000095.1	Yes
	aac(6')-Ib	AAC(6')-Ib family aminoglycoside 6'-N-acetyltransferase	WP_071846335.1	NG_052628.1	OKR93365.1	MPVD01000104.1	No
	aac(6')-Ib	AAC(6')-Ib family aminoglycoside 6'-N-acetyltransferase	WP_087872512.1	NG_067959.1	RNV69984.1	NEYI02000029.1	No
	aac(6')-Ib	AAC(6')-Ib family aminoglycoside 6'-N-acetyltransferase	WP_126123880.1	NG_067960.1	RTB56948.1	RWWW01000055.1	No
	aac(6')-Ib	AAC(6')-Ib family aminoglycoside 6'-N-acetyltransferase	WP_117044474.1	NG_067956.1		UJAY01000171.1	Yes
	aac(6')-Ib	AAC(6')-Ib family aminoglycoside 6'-N-acetyltransferase	WP_148937512.1	NG_067966.1	TYS94893.1	VTBE01000008.1	No
	aac(6')-Ib	AAC(6')-Ib family aminoglycoside 6'-N-acetyltransferase	WP_063840282.1	NG_047285.1		AF012280.1	Yes
aac(6')-Ib3	aac(6')-Ib	aminoglycoside N-acetyltransferase AAC(6')-Ib3	WP_032488579.1	NG_051438.1	AAG33663.1	AY007784.1	No
aac(6')-Ib4	aac(6')-Ib	aminoglycoside N-acetyltransferase AAC(6')-Ib4	WP_003159191.1	NG_051844.1	AAF72941.1	AF231133.1	No
	aac(6')-Ib'	aminoglycoside N-acetyltransferase AAC(6')-Ib'	WP_014454105.1	NG_051695.1	AAT74613.1	AY660529.1	No
	aac(6')-Ib-cr	fluoroquinolone-acetylating aminoglycoside 6'-N-acetyltransferase AAC(6')-Ib-cr	WP_071846215.1	NG_052213.1		KR259315.1	Yes
aac(6')-Ib-cr10	aac(6')-Ib-cr	fluoroquinolone-acetylating aminoglycoside 6'-N-acetyltransferase AAC(6')-Ib-cr10	WP_124042715.1	NG_067968.1		UWWG01000003.1	Yes
aac(6')-Ib-cr11	aac(6')-Ib-cr	fluoroquinolone-acetylating aminoglycoside 6'-N-acetyltransferase AAC(6')-Ib-cr11	WP_159241551.1	NG_067969.1		VDJF01000045.1	Yes

Table S4 (continued). Reference *aac(6′)* gene catalog of the National Database of Antibiotic Resistant Organisms (NDARO), as of 01 July 2024

#Allele	Gene family	Product name	Refseq protein	Refseq nucleotide	Genbank protein	Genbank nucleotide	Curated refseq start
aac(6')-Ib-cr3	aac(6')-Ib-cr	fluoroquinolone-acetylating aminoglycoside 6'-N-acetyltransferase AAC(6')-Ib-cr3	WP_071766621.1	NG_052123.1		HQ170516.1	Yes
aac(6')-Ib-cr4	aac(6')-Ib-cr	fluoroquinolone-acetylating aminoglycoside 6'-N-acetyltransferase AAC(6')-Ib-cr4	WP_065187000.1	NG_052463.1		JZKY01000061.1	Yes
aac(6')-Ib-cr5	aac(6')-Ib-cr	fluoroquinolone-acetylating aminoglycoside 6'-N-acetyltransferase AAC(6')-Ib-cr5	WP_063840321.1	NG_051711.1	ABX24471.1	EU161636.1	No
aac(6')-Ib-cr6	aac(6')-Ib-cr	fluoroquinolone-acetylating aminoglycoside 6'-N-acetyltransferase AAC(6')-Ib-cr6	WP_063840320.1	NG_047292.1		EU675686.2	Yes
aac(6')-Ib-cr7	aac(6')-Ib-cr	fluoroquinolone-acetylating aminoglycoside 6'-N-acetyltransferase AAC(6')-Ib-cr7	WP_071766399.1	NG_052086.1		CP015078.1	Yes
aac(6')-Ib-cr8	aac(6')-Ib-cr	fluoroquinolone-acetylating aminoglycoside 6'-N-acetyltransferase AAC(6')-Ib-cr8	WP_151346866.1	NG_067970.1	AZK52946.1	CP034250.1	No
aac(6')-Ib-cr9	aac(6')-Ib-cr	fluoroquinolone-acetylating aminoglycoside 6'-N-acetyltransferase AAC(6')-Ib-cr9	WP_131534982.1	NG_067971.1	AYD68572.1	MH569711.1	No
	aac(6')-Ib11	aminoglycoside N-acetyltransferase AAC(6')-Ib11	WP_032490438.1	NG_048578.1	AAN41403.1	AY136758.1	No
	aac(6')-Ib11	aminoglycoside N-acetyltransferase AAC(6')-Ib11	WP_069985732.1	NG_052506.1	AEU10765.1	JN412067.1	No
	aac(6')-Id	aminoglycoside N-acetyltransferase AAC(6')-Id	WP_085334908.1	NG_055007.1		X12618.1	Yes
	aac(6')-Ie/ aph(2")	bifunctional AAC(6')-Ie family aminoglycoside N-acetyltransferase/APH(2") family aminoglycoside O-phosphotransferase	WP_122630840.1	NG_062248.1	AUV57982.1	MF547665.1	No
	aac(6')-Ie/ aph(2")-Ia	bifunctional aminoglycoside N-acetyltransferase AAC(6')-Ie/aminoglycoside O-phosphotransferase APH(2")-Ia	WP_032490744.1	NG_047212.1	CAD60196.1	AJ536195.1	No
	aac(6')-Ie/ aph(2")-Ia	bifunctional aminoglycoside N-acetyltransferase AAC(6')-Ie/aminoglycoside O-phosphotransferase APH(2")-Ia	WP_012655884.1	NG_047217.1	BAH18719.1	AP009486.1	No
	aac(6')-Ie/ aph(2")-Ia	bifunctional aminoglycoside N-acetyltransferase AAC(6')-Ie/aminoglycoside O-phosphotransferase APH(2")-Ia	WP_001028144.1	NG_047055.1	AAA88548.1	GU565967.1	No
	aac(6')-Ie/ aph(2")-If2	bifunctional aminoglycoside N-acetyltransferase AAC(6')-Ie2/aminoglycoside O-phosphotransferase APH(2")-If2	WP_057097547.1	NG_062220.1	AGV79342.1	KF652095.1	No
	aac(6')-If	aminoglycoside N-acetyltransferase AAC(6')-If	WP_048233550.1	NG_052054.1	KLV54643.1	LESO01000005.1	No
	aac(6')-If	aminoglycoside N-acetyltransferase AAC(6')-If	WP_071593229.1	NG_047295.2		X55353.1	Yes
	aac(6')-Il	aminoglycoside N-acetyltransferase AAC(6')-Il	WP_063840324.1	NG_047304.1	ACY07912.1	GQ856540.1	No
	aac(6')-Il	aminoglycoside N-acetyltransferase AAC(6')-Il	WP_063840325.1	NG_047305.1	ADO50680.1	HM750249.1	No
	aac(6')-Il	aminoglycoside N-acetyltransferase AAC(6')-Il	WP_013263788.1	NG_047303.1	AAA90937.1	U13880.2	No
	aac(6')-Il	aminoglycoside N-acetyltransferase AAC(6')-Il	WP_071846318.1	NG_052392.1	AGH30448.1	KC597709.1	No
	aac(6')-Im	aminoglycoside N-acetyltransferase AAC(6')-Im	WP_010708502.1	NG_047306.1	AAK63041.1	AF337947.1	No
	aac(6')-Im	aminoglycoside N-acetyltransferase AAC(6')-Im	WP_002592614.1	NG_052530.1	ENZ04101.1	AGYO01000035.1	No
	aac(6')-Im	aminoglycoside N-acetyltransferase AAC(6')-Im	WP_057038955.1	NG_052222.1	KWV61377.1	LKIV02000117.1	No
	aac(6')-Ip	aminoglycoside 6'-N-acetyltransferase AAC(6')-Ip	WP_069453146.1	NG_047307.2		Z54241.1	Yes
	aac(6')-Iq	aminoglycoside 6'-N-acetyltransferase AAC(6')-Iq	WP_032489188.1	NG_047308.1	AAC25500.1	AF047556.1	No
	aac(6')-Iz	aminoglycoside N-acetyltransferase AAC(6')-Iz	WP_012481011.1	NG_052343.1	BAP12403.1	AB971834.1	No
	aac(6')-Iz	aminoglycoside N-acetyltransferase AAC(6')-Iz	WP_005414154.1	NG_052390.1	EMF61681.1	AMXM01000008.1	No
	aac(6')-Iz	aminoglycoside N-acetyltransferase AAC(6')-Iz	WP_024956926.1	NG_052348.1	AIL09067.1	CP008838.1	No
	aac(6')-Iz	aminoglycoside N-acetyltransferase AAC(6')-Iz	WP_071846310.1	NG_052377.1	ADQ43423.1	HQ424462.1	No
	aac(6')-Iz	aminoglycoside N-acetyltransferase AAC(6')-Iz	WP_005410660.1	NG_047317.1	AAD52985.1	AF140221.1	No
	aac(6')-Iz	aminoglycoside N-acetyltransferase AAC(6')-Iz	WP_049440615.1	NG_054659.1		JVWJ01000294.1	Yes
	aac(6')-kana	aminoglycoside 6'-N-acetyltransferase	WP_055545096.1	NG_052084.1		LIQU01000069.1	Yes

Table S5. Curation of the NDARO Reference Gene Catalog for *aac(6´)-Ib*-like amino acid sequence homologies.

aac(6´)-Ib amino acid sequences (W87, Q101, L102, D164)		***aac(6´)-Ib***$_{4}$ amino acid seqences (W87, Q101, S102, D164)		***aac(6´)-Ib***$_{11}$ amino acid sequences (W87, L101, S102, D164)		***aac(6´)-Ib-cr*** amino acid sequences (R87, Q101, L102, Y164)	
**Refseq protein**	**Gene family as per NDARO**	**Refseq protein**	**Gene family as per NDARO**	**Refseq protein**	**Gene family as per NDARO**	**Refseq protein**	**Gene family as per NDARO**
WP_063840283.1	*aac(6´)-Ib*	WP_071846239.1	*aac(6´)*	WP_065187201.1	*aac(6´)-Ib*	WP_071846332.1	*aac(6´)-Ib*
WP_063978482.1	*aac(6´)-Ib*	WP_071846254.1	*aac(6´)-Ib*	WP_032490438.1	*aac(6´)-Ib*$_{11}$	WP_085844237.1	*aac(6´)-Ib*
WP_063840319.1	*aac(6´)-Ib*	WP_071846301.1	*aac(6´)-Ib*	WP_069985732.1	*aac(6´)-Ib*$_{11}$	WP_117046752.1	*aac(6´)-Ib*
WP_101516660.1	*aac(6´)-Ib*	WP_071593232.1	*aac(6´)-Ib*			WP_071846215.1	*aac(6´)-Ib-cr*
WP_085843736.1	*aac(6´)-Ib*	WP_013250882.1	*aac(6´)-Ib*			WP_071766621.1	*aac(6´)-Ib-cr3*
WP_063612062.1	*aac(6´)-Ib*	WP_134628915.1	*aac(6´)-Ib*			WP_065187000.1	*aac(6´)-Ib-cr4*
WP_071846208.1	*aac(6´)-Ib*	WP_071984682.1	*aac(6´)-Ib*			WP_063840321.1	*aac(6´)-Ib-cr5*
WP_071846209.1	*aac(6´)-Ib*	WP_104442471.1	*aac(6´)-Ib*			WP_063840320.1	*aac(6´)-Ib-cr6*
WP_071846373.1	*aac(6´)-Ib*	WP_151405943.1	*aac(6´)-Ib*			WP_071766399.1	*aac(6´)-Ib-cr7*
WP_071846241.1	*aac(6´)-Ib*	WP_071846361.1	*aac(6´)-Ib*			WP_151346866.1	*aac(6´)-Ib-cr8*
WP_064765190.1	*aac(6´)-Ib*	WP_071846382.1	*aac(6´)-Ib*			WP_131534982.1	*aac(6´)-Ib-cr9*
WP_071593230.1	*aac(6´)-Ib*	WP_071846385.1	*aac(6´)-Ib*			WP_124042715.1	*aac(6´)-Ib-cr10*
WP_063840280.1	*aac(6´)-Ib*	WP_071846249.1	*aac(6´)-Ib*			WP_159241551.1	*aac(6´)-Ib-cr11*
WP_069067473.1	*aac(6´)-Ib*	WP_071846291.1	*aac(6´)-Ib*				
WP_063840281.1	*aac(6´)-Ib*	WP_022631510.1	*aac(6´)-Ib*				
WP_159287722.1	*aac(6´)-Ib*	WP_071846329.1	*aac(6´)-Ib*				
WP_113613509.1	*aac(6´)-Ib*	WP_071593200.1	*aac(6´)-Ib*				
WP_123085936.1	*aac(6´)-Ib*	WP_071846262.1	*aac(6´)-Ib*				
WP_079452738.1	*aac(6´)-Ib*	WP_071846281.1	*aac(6´)-Ib*				
WP_132629969.1	*aac(6´)-Ib*	WP_012695458.1	*aac(6´)-Ib*				
WP_140423191.1	*aac(6´)-Ib*	WP_072201103.1	*aac(6´)-Ib*				
WP_022631162.1	*aac(6´)-Ib*	WP_071846335.1	*aac(6´)-Ib*				
		WP_087872512.1	*aac(6´)-Ib*				
		WP_126123880.1	*aac(6´)-Ib*				
		WP_117044474.1	*aac(6´)-Ib*				
		WP_148937512.1	*aac(6´)-Ib*				
		WP_003159191.1	aac(6´)-Ib$_{4}$				
		WP_014454105.1	aac(6´)-Ib'				

aac(6´)-Ib$_{4}$******* amino acid seq. (R87, Q101, S102, D164)	
**Refseq protein**	**Gene family as per NDARO**
WP_117065919.1	*aac(6´)-Ib*

In the NDARO, many of the individual RefSeqs are simply assigned to aac(6´)-Ib without further subclassification. Hence, amino acid sequences for these were aligned to identify sequence alterations that allowed assignment to one of the four sub-classifications displayed above (Fig. S2). RefSeq WP_117065919.1 represents a previously undescribed W87R variant of aac(6´)-Ib$_{4}$.

Table S6. Engineered *E. coli* strains constructed in this study

Strain	RefSeq ORF	Gene name		Phenotype	Annotations in the NDARO (*n*)			
		NDARO	This Study		*Enterob.*	*P. aer.*	*A. baum.*	Gram-pos.
EC145	WP_063840321	*aac(6')-Ib-cr*	*aac(6')-Ib-cr*	AAC(6')-I	34123	75	18	0
EC144	WP_063840280	*aac(6')-Ib*	*aac(6')-Ib*	AAC(6')-I	14372	1187	378	25
EC358	WP_013250882	*aac(6')-Ib*	*aac(6')-Ib* $_4$	AAC(6')-II	4250	2247	4434	0
EC330	WP_013263788	*aac(6')-Il*	*aac(6')-Il*	AAC(6')-I	867	1195	3	0
EC149	WP_023622803	*aac(6')-IIa*	*aac(6')-IIa*	AAC(6')-II	1460	456	12	1
EC329	WP_064190969	*aac(6')-29*	*aac(6')-29*	AAC(6')-I	23	591	0	0
EC146	WP_033649026	*aac(6')-Ic*	*aac(6')-Ic*	AAC(6')-I	297	0	0	0
EC365	WP_071593229	*aac(6')-If*	*aac(6')-If*	AAC(6')-I	134	0	0	0
EC345	WP_065187201	*aac(6')-Ib*	*aac(6')-Ib* $_{11}$	AAC(6')-IV	53	0	1	0
EC143	WP_001028144	*aac(6')-Ie*	*aac(6')-Ie*	AAC(6')-I'	18	1	0	22591
EC334	WP_005410660	*aac(6')-Iz*	*aac(6')-Iz*	AAC(6')-I	1	0	0	0
EC148	WP_008265821	*aac(6')-Ii*	*aac(6')-Ii*	AAC(6')-III	0	0	0	23
EC332	WP_016541245	*aac(6')-Ih*	*aac(6')-Ih*	AAC(6')-I	0	0	0	0
EC328	WP_030403494	*aac(6')*	*aac(6')-Isa*	AAC(6')-II	0	0	0	0
EC343	WP_012695484	*aac(6')-IIc*	*aac(6')-IIc*	AAC(6')-II	0	0	0	0
EC366	WP_038790798	*aac(6')-III*	*aac(6')-III*	AAC(6')-III	0	0	0	0
EC333	WP_049646085	*aac(6')*	*aac(6')-Y*	AAC(6')-III	0	0	0	0
